# The profiling and analysis of gene expression in human periodontal ligament tissue and fibroblasts

**DOI:** 10.1002/cre2.533

**Published:** 2022-02-01

**Authors:** Nattakarn Hosiriluck, Haruna Kashio, Ayuko Takada, Itaru Mizuguchi, Toshiya Arakawa

**Affiliations:** ^1^ Division of Biochemistry, Department of Oral Biology, School of Dentistry Health Sciences University of Hokkaido Tobetsu‐cho Hokkaido Japan; ^2^ Division of Orthodontics and Dentofacial Orthopedics, Department of Oral Growth and Development, School of Dentistry Health Sciences University of Hokkaido Tobetsu‐cho Hokkaido Japan; ^3^ Department of Orthodontics and Dentofacial Orthopedics, Graduate School of Dentistry Tohoku University Sendai Japan; ^4^ Present address: Nattakarn Hosiriluck, Department of Masticatory Sciences (15th floor), Faculty of Dentistry Mahidol University No. 6, Yothi Road, Ratchathewi District Bangkok 10400 Thailand

**Keywords:** extracellular matrixes, fibroblasts, periodontal ligament, profiling of gene expression

## Abstract

**Objectives:**

The periodontal ligament (PDL) is an important component of periodontium to support dental structure in the alveolar socket. Regeneration of PDL tissue is an effective treatment option for periodontal disease and the profiling of genes involved in this process will be informative. Therefore, our study aims to accurately delineate the profiling of gene expression for PDL tissue regeneration.

**Materials and Methods:**

We isolated PDL tissues and PDL fibroblasts (PDLFs) from premolar teeth, which were extracted from healthy periodontal status patients undergoing orthodontic treatment. Messenger RNA (mRNA) expression in PDL tissue and PDLFs were analyzed using Cap analysis gene expression, which is a second‐generation sequencing technique to create profiling. We also determined the protein expression using Western blot.

**Results:**

Collagens (type I, III, and VI), noncollagenous proteins (periostin and osteonectin), and proteoglycans (asporin, lumican, decorin, and osteomodulin) were highly expressed in PDL tissue. Integrin, β1 was also expressed in PDL tissue. On comparison of gene expression between PDL tissue and PDLFs, four PDL marker genes, osteopontin, asporin, periostin, and osteonectin, were decreased in PDLFs. The genes for gene regulation were also highly expressed.

**Conclusions:**

Our study demonstrated the overall profiling of mRNA expression in PDL tissue and analyzed the important genes which may be useful for providing specific information for the reconstruction of PDL. We also identified the difference in gene expression between PDL tissue and PDLFs which might provide insights towards PDL regeneration.

## 1 INTRODUCTION

Periodontium is a critical complex tissue that consists of gingiva, cementum, alveolar bone, and periodontal ligament (PDL). PDL connects the tooth into the alveolar socket by maintaining the attachment between the dental root and bone surface, and provides a cushion for occlusal loading in the masticatory system. Moreover, PDL is able to remodel itself, responding to mechanical stress and maintaining its structure and function under physiologic conditions (Chukkapalli & Lele, 2018; Smith et al. 2019). From a pathological perspective, mechanical overloading may result in the destruction of PDL structure. In current clinical practice, scaling and root planing are commonly performed. However, these treatments are not applicable in severed periodontitis status patients. Thus, PDL regeneration is required as a new treatment option.

Extracellular matrixes (ECMs) are considered as key factors for the tissue repair. In previous studies, collagens, proteoglycans, and noncollagenous proteins were identified as the key ECMs of PDL, (Berkovitz, 1990; Butler et al., 1975; Dublet et al., 1988; Embery, 1990) and most studies had focused on identifying genes from PDL fibroblasts (PDLFs) instead of tissue because PDLFs are the most abundant cells in PDL tissue. Collagen type I is the major collagen to form the main structure of PDL fiber while other types such as type III are codistributed with collagen type I. Proteoglycans (e.g., asporin, decorin, and versican (VCAN; Larjava et al., 1992; Yamada et al., 2006) and noncollagenous proteins (e.g., periostin; Xu et al., 2017) have also been reported in PDLFs. However, little is known about the overall profiling of gene expression in PDL tissue. According to a comparison of gene expression between PDLFs and PDL tissue, osteopontin was significantly high in PDL tissue (Lallier & Spencer, 2007). Other genes such as pleiotrophin, osteomodulin, alkaline phosphatase, bone sialoprotein 2, periostin, and fibromodulin (FMOD) were slightly high in PDL tissue as well. Based on these results, there has been speculation that the cultured PDLFs may represent an immature cellular form of PDLFs (Lallier & Spencer 2007; Marchesan et al., 2011). Based on these results, there has been speculation that the cultured PDLFs in vitro may present a different nature compared from the PDLFs in PDL tissue under in vivo. These different cell conditions results the different gene expression. Thus, culturing PDLFs from PDL tissue may alter the ECMs properties due to the environmental changes from in vivo to in vitro. To regenerate PDL tissue using PDLFs, recovering the originated ECMs in vivo must also be considered. Furthermore, epithelial cell rest of Malassez, blood vessel, and lymphatic vessel are also present in the PDL tissue area. Including, osteoclasts, osteoblasts, and cementoblasts are located in the hard tissue surface. These components may provide molecules such as ECMs in PDL tissue to create a structure and support function. However, the gene profiling only from PDLFs in culture may be altered from the environmental change and cause insufficient data for the regeneration of PDL tissues. Thus, gene expression in PDL tissue may include genes that are derived from these cells and it may be critical to identify the overall gene expression pattern in PDL tissue. The information of overall gene expression in PDL tissue may be necessary for PDL tissue regeneration.

Our study is the first study to profile and analyze gene expression of PDL tissue, using Cap analysis gene expression (CAGE) method. We analyzed (1) the profiling of whole messenger RNA (mRNA) expression of PDL tissue by second generation sequencing, (2) the comparison of mRNA expression between PDL tissue and PDLFs from the same patient samples.

## 2 MATERIALS AND METHODS

### 2.1 Sample information

PDL tissue was collected from eight patients who were required for premolars extraction for orthodontic treatment, purposes under permission by the Ethics Committee, Health Sciences University of Hokkaido (Permission No. 135). Our study included only sound teeth with healthy periodontal status. Other conditions such as caries, periodontitis, endodontic treated as well as an unerupted condition must be excluded. The female patients, age 12–26 years old, were included for this study and all of them are nonsmokers. Their identification information has been acquired for this study including, but not limited to, initials, hospital numbers, medical history, surgical history, and dental history. All the information was kept strictly confidential. The patients were deidentified into the code numbers specifically for this study. The patients' individual information was neither utilized for any purpose other than the purpose of the study nor published in this manuscript. The collected sample from the patients were applied to different methods (Table S1).

### 2.2 Materials

Minimum essential medium Eagle alpha modification (α‐MEM) and fetal bovine serum (FBS) were provided from Sigma‐Aldrich Co., Bambanker™ from Nippon Genetics, Trypsin from Gibco, RNeasy Mini Kit from Qiagen, TRIzol™ reagent and reverse transcription system, from Invirtogen™, Ex Taq™ from TaKaRa Co. Primers were provided from Hokkaido System Science Co., Ltd. and antibodies were obtained from Abcam (integrin β1: ab52971, integrin α5: ab150361, osteopontin: ab91655), GeneTex (asporin: GTX104790, periostin: GTX100602, osteonectin: GTX133747, collagen type6A1: GTX109963) and Sigma‐Aldrich Co., (beta‐actin: AC‐15), using 1:1000 dilution.

### 2.3 PDLFs isolation from PDL tissue

PDL tissue was collected from teeth that were extracted for orthodontic treatment purposes. The extracted teeth were rinsed with 10% penicillin–streptomycin‐added phosphate‐buffered saline. After rinsing, PDL tissue was removed from a tooth at the middle part of the dental root using a surgical scalpel blade (No. 11). For PDLFs, the PDL tissue was applied directly to a collagen‐coated culture flask that contained 1 ml culture medium then the flask was flipped upside‐down for 1–2 h which caused PDL tissue to attach on the cultured surface of the flask. After the attachment of PDL tissue, the culture flask was flipped back and 3 ml culture medium was added.

### 2.4 PDLFs culture

The isolated PDL tissue was cultured in α‐MEM with 10% FBS, penicillin/streptomycin (1 mg/ml), 1% glutamine and amphotericin B (2. 5μg/ml) at 37°C with 5% CO_2_. When outgrown to 80% confluence, PDLFs were subcultured and frozen with Bambanker™ as a stock at 1 × 10^5^ cells/ml. PDLFs at passages 2–4 were used for experiments. PDLFs were cultured in α‐MEM with 10% FBS, penicillin/streptomycin (1 mg/ml), 1% glutamine and amphotericin B (2.5 μg/ml) at 37°C with 5% CO_2_.

### 2.5 RNA isolation

RNA was isolated from PDL tissue using 1 ml of TRIzol reagent and homogenized with a homogenizer under instruction. For PDLFs, RNA was isolated using Qiagen, RNeasy Mini Kit. Both RNAs were solubilized with RNase‐free water and stored at −80°C. The RNA concentration was measured with NanoDrop™ 1000 (Thermo Fisher Scientific).

### 2.6 Cap analysis gene expression

CAGE, a second‐generation sequencing technique that produced a short nucleotide sequence (50 nucleotides) from the 5ʹ end of mRNA to determine the gene expression from the sample, operated by DNAFORM Co. CAGE was applied to PDL tissue and PDLFs from four patients. Patient nos. 1 and 2 provided both PDL tissue and PDLFs while Patient no. 3 provided only PDL tissue and Patient no. 4 provided only PDLFs. The average of gene expression was calculated and measured in counts per million.

### 2.7 Polymerase chain reaction

Primers of four PDL markers (osteopontin, asporin, periostin, and osteonectin) and GAPDH were designed using Primer‐BLAST and Primer 3 Plus program as shown in Table 1. RNA samples (1 μg) were converted to complementary DNA (cDNA) using the SuperScript™ II Reverse Transcriptase System. For conventional polymerase chain reaction (PCR), cDNA (1 μl) were utilized with specific primers and TaKaRa Ex Taq™ for 35 cycles. The PCR products were analyzed by agarose gel electrophoresis. DNA bands were captured with Light‐Captured II, Cooled CCD Camera System. For quantitative PCR (qPCR), cDNA (1 μl) was utilized with specific primers, using KAPA SYBR® FAST qPCR Master Mix (2X) Kit. The $2^{‐∆∆C_{t}}$ value was calculated for fold change of gene expression.

**Table 1:** Primer sets of four PDL markers and GAPDH

Gene	Primer sequence		Product size (bp)
OPN (NM_001040058.2)	Forward	ACCCATCTCAGAAGCAGAATCTCC	462
	Reverse	CACCATTCAACTCCTCGCTTTCC	
ASPN (NM_017680.5)	Forward	CTTTGTGCTCTGCCAAACCC	440
	Reverse	GGACAGATACAGCCTTCGCA	
POSTN (NM_006475.3)	Forward	GTCTTTGAGACGCTGGAAGG	201
	Reverse	CAAGATCCGTGAAGGTGGTT	
SPARC (NM_003118.4)	Forward	GGAAGAAACTGTGGCAGAGGTGA	469
	Reverse	TGTTGTCCTCATCCCTCTCATAC	
GAPDH (NM_002046.7)	Forward	GAGAAGGCTGGGGCTCATTT	231
	Reverse	AGTGATGGCATGGACTGTGG	

Abbreviations: bp, base pair; PDL, periodontal ligament.

### 2.8 Protein detection by Western blot

Protein concentrations were measured by spectrophotometry, GeneQuant Pro., using XL‐Bradford (APRO SCIENCE). Protein samples (10 μg) were separated using 5%–20% sodium dodecyl sulfate–polyacrylamide gel electrophoresis gel and transferred to polyvinylidene difluoride membranes. Membranes were blocked with 10% blockA‐PBST overnight and incubated with primary antibody for 2 h. The protein bands were detected using enhanced chemiluminescence, Immobilon Western Chemiluminescent HRP Substrate (Millipore), and captured with Light‐Captured II, Cooled CCD Camera System.

## 3 RESULTS

### 3.1 Profiling of gene expression in PDL tissue

#### 3.1.1 Top 20 gene expression in PDL tissue and PDLFs

**Figure 1:**
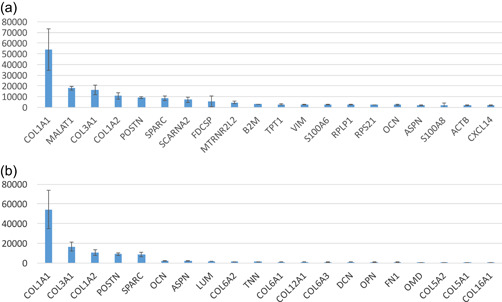
CAGE data reported (a) the expression level of top 20 most expressed genes in PDL tissue (*Y*‐axis shows the expression level as counts per millions [cpm]) and (b) important ECM genes that are highly expressed in PDL tissue (*Y*‐axis refers to gene expression level [cpm]). CAGE, Cap analysis gene expression; ECM, extracellular matrix; PDL, periodontal ligament

**Figure 2:**
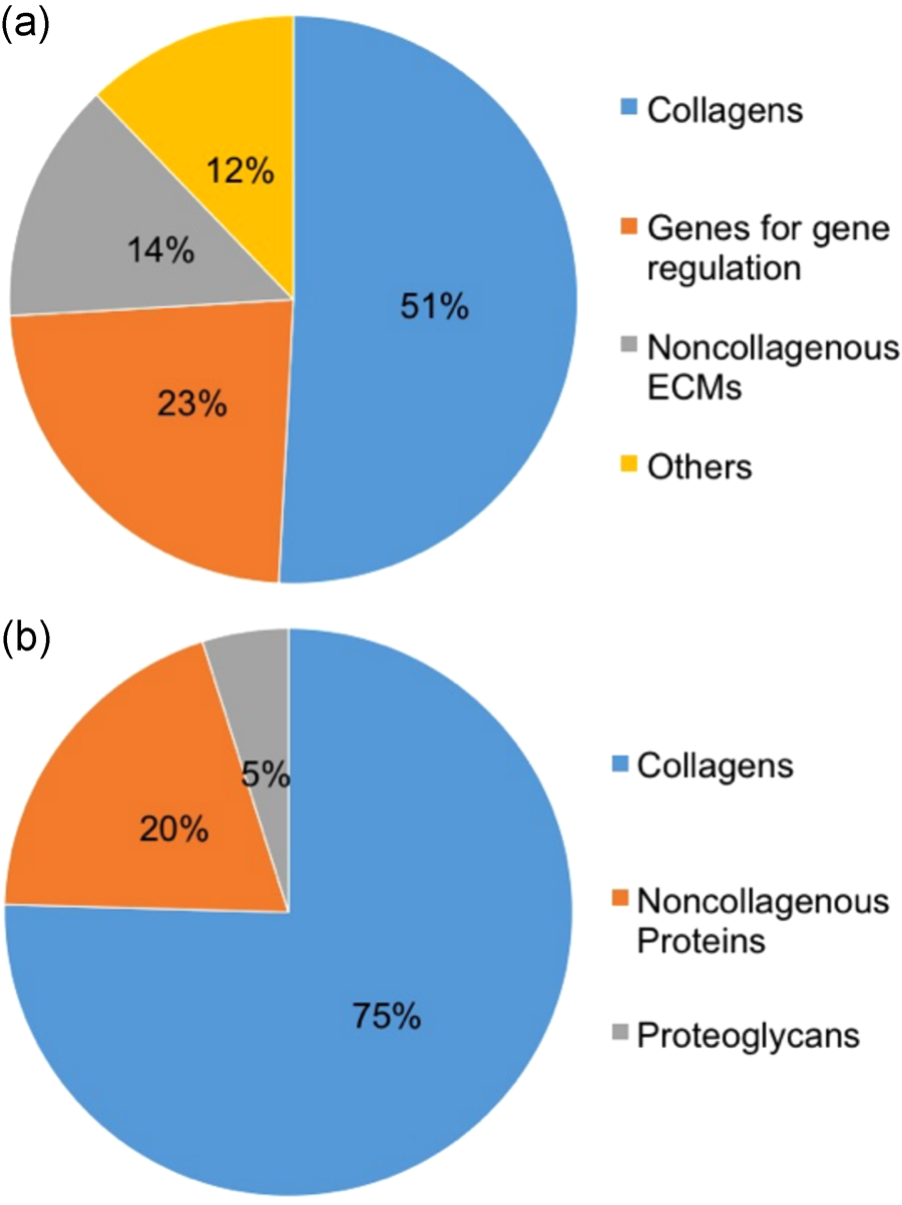
The summary of gene profiling by categories in PDL tissue. (a) Ratio of four categories in all top 20 genes: collagens (51%), genes for gene regulation (23%), noncollagenous ECM (14%), and others (12%). (b) Ratio of three categories in top 20 ECM genes: collagens (75%), noncollagenous proteins (20%), and proteoglycans (5%). ECM, extracellular matrix; PDL, periodontal ligament

The top 20 most highly expressed genes in PDL tissue by the CAGE method are shown in Figure 1a and Table 2. In PDL tissue, these genes were able to be categorized into four groups: collagens (50.82%), genes for gene regulation (23.27%), noncollagenous ECM (13.73%), and others (12.18%), respectively, as shown in Figure 2a. In collagen, collagen type I α1 (COL1A1: 33.77%), type III α1 (COL3A1: 10.35%), and type I α2 (COL1A2: 6.69%) were highly expressed in PDL tissue. Genes for gene regulation in PDL tissue were metastasis‐associated lung adenocarcinoma transcript 1 (MALAT1: 11.16%), small Cajal body‐specific RNA (SCARNA2: 4.52%), humanin‐like2 (MTRNR2L2: 2.92%), translationally controlled tumor protein (TPT1: 1.72%), ribosomal protein lateral stalk subunit P1 (RPLP1: 1.51%), and ribosomal protein S21 (RPS21: 1.44%). Noncollagenous ECM were periostin (POSTN: 5.67%), osteonectin (SPARC: 5.31%), osteocalcin (OCN: 1.44%), and asporin (ASPN: 1.31%). Other highly expressed genes in PDL tissue were follicular dendritic cell secreted protein (FDC‐SP: 3.39%), *beta‐2‐microglobulin* (B2M: 1.83%), vimentin (VIM: 1.70%), S100 calcium‐binding protein A6 (S100A6: 1.55%), S100 calcium‐binding protein A8 (S100A8: 1.28%), beta‐actin (ACTB: 1.23%), and chemokine (C‐X‐C motif) ligand 14 (CXCL14: 1.18%), respectively. In PDLFs (Table 2), highly expressed genes were different from PDL tissue which were COL1A1 (15.88%), S100A6 (S100A6:9.72%), MALAT1 (8.87%), actin beta (ACTB: 8.4%), ferritin heavy chain 1 (FTH1: 6.7%).

**Table 2:** Percentage of top 20 genes in PDL tissue and PDLFs

Top 20 genes in PDL tissue	Percentage	Top 20 genes in PDLFs	Percentage
COL1A1	33.77	COL1A1	15.88
MALAT1	11.16	S100A6	9.72
COL3A1	10.35	MALAT1	8.87
COL1A2	6.69	ACTB	8.40
POSTN	5.67	FTH1	6.70
SPARC	5.31	TMSB10	5.58
SCARNA2	4.52	TMSB4X	4.67
FDCSP	3.39	VIM	4.25
MTRNR2L2	2.92	ACTG1	4.15
B2M	1.83	COL1A2	3.46
TPT1	1.72	TPT1	3.38
VIM	1.70	RPLP1	3.15
S100A6	1.55	GAPDH	3.11
RPLP1	1.51	LGALS1	2.92
RPS21	1.44	MYL6	2.79
OCN	1.44	MYL6B	2.73
ASPN	1.31	RP11	2.72
S100A8	1.28	MTRNR2L2	2.53
ACTB	1.23	RPS21	2.50
CXCL14	1.18	FTL	2.47

Abbreviations: PDL, periodontal ligament; PDLF, PDL fibroblast.

#### 3.1.2 Top 20 highly expressed ECM genes in PDL tissue

In Figures 1b and 2b, highly expressed ECMs in PDL tissue were able to be categorized in three groups: collagens (75.44%: type I, III, VI, V, XII, and XVI, respectively), noncollagenous proteins (19.66%: POSTN, SPARC, OCN, tenascin‐N [TNN], osteopontin [OPN], fibronectin [FN1]), and proteoglycans (4.90%: ASPN, lumican [LUM], decorin [DCN], osteomodulin [OMD]).

### 3.2 Profiling of gene expression in each category of ECMs

#### 3.2.1 Collagens

**Figure 3:**
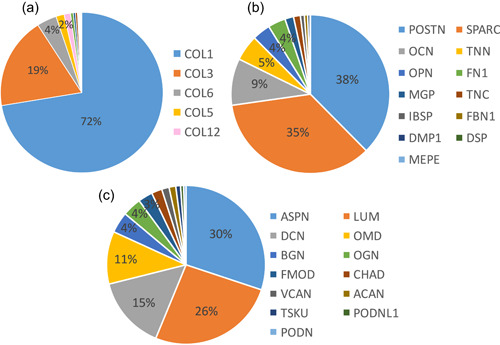
The summary of gene profiling of collagens, noncollagenous proteins, and proteoglycans in PDL tissue. (a) The ratio of collagens in PDL tissue mainly were collagen type I (72%) and III (19%), followed by type VI (4%), V (2%), XII (1%), and others. (b) Ratio of noncollagenous proteins in PDL tissue mainly were POSTN (38%) and SPARC (35%). (c) Ratio of proteoglycan in PDL tissue mainly were ASPN (30%), LUM (26%), DCN (15%), and OMD (11%). ASPN, asporin; DCN, decorin; LUM, lumican; OMD, osteomodulin; PDL, periodontal ligament; POSTN, periostin

In PDL tissue, 27 types of collagens were found to exist. Collagen type I (72.40%: COL1A1 [60.42%], COL1A2 [11.97%]) was the major subtype and type III (18.52%: COL3A1) was the second, followed by collagen type VI (3.94%: COL6A1 [1.28%], COL6A2 [1.43%], COL6A3 [1.23%]), V (1.62%: COL5A1 [0.76%], COL5A2 [0.77%], COL5A3 [0.09%]), and XII (1.27%: COL12A1), respectively, as shown in Figure 3a and Table 3a,b. These collagens are able to be categorized by the function which are fibril‐forming collagen (type I, III, and V), microfibrillar collagen (type VI), and fibril‐associated collagens with interrupted triple helices (FACIT collagen: type XII).

**Table 3a:** Types of collagens in PDL tissue

No.	Collagen type	Percentage	No.	Collagen type	Percentage
1	COL1	72.40	15	COL17	0.05
2	COL3	18.52	16	COL8	0.05
3	COL6	3.94	17	COL27	0.05
4	COL5	1.62	18	COL9	0.04
5	COL12	1.27	19	COL24	0.03
6	COL16	0.56	20	COL10	0.02
7	COL11	0.43	21	COL26	0.01
8	COL4	0.39	22	COL25	0.01
9	COL18	0.14	23	COL28	0.01
10	COL13	0.11	24	COL23	0.004
11	COL21	0.11	25	COL22	0.004
12	COL14	0.09	26	COL2	0.004
13	COL7	0.09	27	COL20	0.001
14	COL15	0.06			

Abbreviation: PDL, periodontal ligament.

**Table 3b:** All collagens in PDL tissue

Collagens	Percentage	Collagens	Percentage
COL1A1	60.42	COL17A1	0.05
COL3A1	18.52	COL27A1	0.05
COL1A2	11.97	COL4A3BP	0.03
COL6A2	1.43	COL24A1	0.03
COL6A1	1.28	COL8A2	0.03
COL12A1	1.27	COL9A2	0.02
COL6A3	1.23	COL10A1	0.02
COL5A2	0.77	COL8A1	0.02
COL5A1	0.76	COL9A3	0.01
COL16A1	0.56	COL26A1	0.01
COL11A1	0.42	COL25A1	0.01
COL4A1	0.18	COL4A5	0.01
COL4A2	0.17	COL28A1	0.01
COL18A1	0.14	COL23A1	0.004
COL13A1	0.11	COL11A2	0.004
COL21A1	0.11	COL2A1	0.004
COL14A1	0.09	COL22A1	0.004
COL5A3	0.09	COL20A1	0.001
COL7A1	0.08	COL4A4	0.001
COL15A1	0.06		

Abbreviation: PDL, periodontal ligament.

#### 3.2.2 Noncollagenous proteins

As shown in Figure 3b and Table 4, POSTN (37.59%) and SPARC (35.16%) were the two most highly expressed noncollagenous proteins in PDL tissue. Others were OCN (9.54%), TNN (5.27%), OPN (3.68%), FN1 (3.56%), matrix gla protein (1.78%), tenascin‐C (1.39%), bone sialoprotein (0.81%), fibrillin‐1 (0.66), dentin matrix acidic phosphoprotein 1 (0.48%), desmoplakin (0.06%), and matrix extracellular phosphoglycoprotein (0.02%).

**Table 4:** Noncollagenous proteins in PDL tissue

Noncollagenous proteins	Percentage
POSTN	37.59
SPARC	35.16
OCN	9.54
TNN	5.27
OPN	3.68
FN1	3.56
MGP	1.78
TNC	1.39
IBSP	0.81
FBN1	0.66
DMP1	0.48
DSP	0.06
MEPE	0.02

Abbreviation: PDL, periodontal ligament.

#### 3.2.3 Proteoglycans

ASPN (30.10%), LUM (26.12%), DCN (14.87%), and OMD (10.74%) were four major proteoglycans in PDL tissue, as shown in Figure 3c and Table 5. Others were biglycan (BGN; 4.27%), osteoglycin (3.85%), FMOD (2.96%), chondroadherin (2.14%), VCAN (1.51%), aggrecan (1.36%), tsukushi (0.97%), podocan‐like protein 1 (0.63%), and podocan (0.49%)

**Table 5:** Proteoglycans in PDL tissue

Proteoglycans	Percentage
ASPN	30.10
LUM	26.12
DCN	14.87
OMD	10.74
BGN	4.27
OGN	3.85
FMOD	2.96
CHAD	2.14
VCAN	1.51
ACAN	1.36
TSKU	0.97
PODNL1	0.63
PODN	0.49

Abbreviation: PDL, periodontal ligament.

### 3.3 Comparison of gene expression between PDL tissue and PDLFs

#### 3.3.1 ECMs

**Figure 4:**
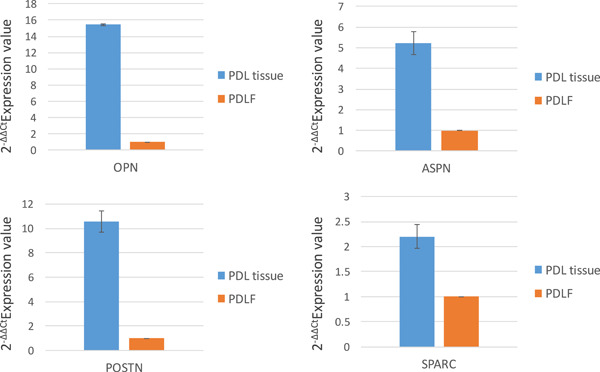
Comparison of gene expression between PDL tissue and PDLFs by quantitative PCR in four PDL marker genes (OPN, ASPN, POSTN, and SPARC). PCR, polymerase chain reaction; PDL, periodontal ligament; PDLF, PDL fibroblast

Comparison of the profiling patterns of PDL tissue and PDLFs from two patients shows that many ECM genes were decreased in PDLFs compared to PDL tissue. The decreased genes included four PDL markers, OPN, POSTN, ASPN, and SPARC, as shown in Tables 6 and 7. Quantitative determinations of the four PDL markers confirmed these results, as shown in Figure 4.

**Table 6:** Expression of ECM genes in PDL tissue and PDLFs of Patient 1

Gene	PDL tissue	PDLFs	Fold change
TNN	1320.48	0.08	16,506.00
OPN	855.48	0.08	10,693.50
OCN	1529.36	0.20	7646.80
OMD	663.68	0.88	754.18
ASPN	1714.12	2.84	603.56
POSTN	6969.29	187.12	37.25
LUM	1847.72	71.28	25.92
COL3A1	14,993.11	1416.92	10.58
COL12A1	1144.68	192.84	5.94
COL16A1	372.40	92.16	4.04
COL5A1	411.44	205.44	2.00
SPARC	5617.65	3121.61	1.80
COL1A2	8218.90	5108.33	1.61
COL1A1	40,956.32	26,045.81	1.57
COL6A3	1057.44	718.52	1.47
DCN	886.48	621.44	1.43
COL5A2	518.40	596.60	0.87
COL6A2	1049.96	1864.96	0.56
FN1	772.84	1752.60	0.44
COL6A1	1155.00	2816.69	0.41

Abbreviations: ECM, extracellular matrix; PDL, periodontal ligament; PDLF, PDL fibroblast.

**Table 7:** Expression of ECM genes in PDL tissue and PDLFs of Patient 2

Gene	PDL tissue	PDLFs	Fold change
TNN	1058.64	0.12	8822.00
OPN	1425.16	0.17	8383.29
OCN	3122.53	0.75	4163.37
OMD	871.40	33.94	25.67
POSTN	10,789.34	617.24	17.48
ASPN	2533.45	174.80	14.49
COL3A1	9815.38	1043.93	9.40
LUM	1670.60	239.27	6.98
COL16A1	353.08	64.76	5.45
COL5A1	856.88	316.78	2.70
SPARC	6827.89	4209.33	1.62
COL6A3	734.36	478.09	1.54
COL5A2	628.72	424.49	1.48
COL1A1	29,131.86	22,852.53	1.27
COL1A2	7665.50	6315.72	1.21
COL12A1	726.52	681.38	1.07
DCN	1089.04	1480.45	0.74
COL6A2	1144.04	2259.99	0.51
COL6A1	840.80	2968.94	0.28
FN1	1063.04	5678.03	0.19

Abbreviations: ECM, extracellular matrix; PDL, periodontal ligament; PDLF, PDL fibroblast.

#### 3.3.2 Integrins

**Figure 5:**
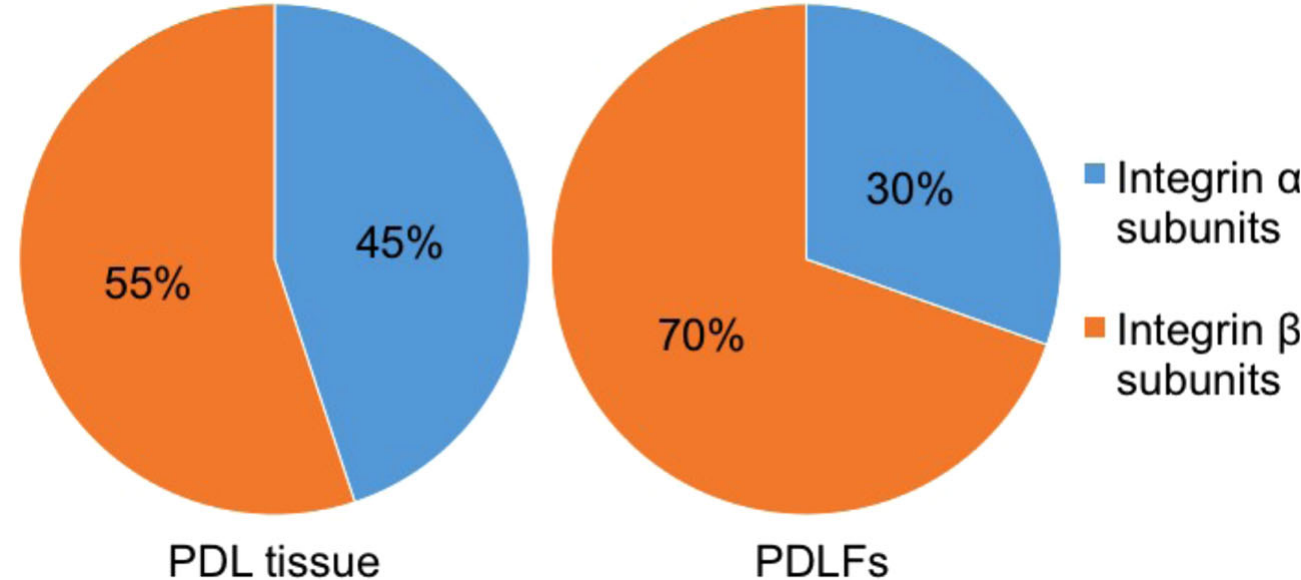
The ratio of integrin α and β in PDL tissue and PDLFs. Integrin α and β were equally expressed in PDL tissue while integrin β was relatively highly expressed in PDLFs. PDL, periodontal ligament; PDLF, PDL fibroblast

**Figure 6:**
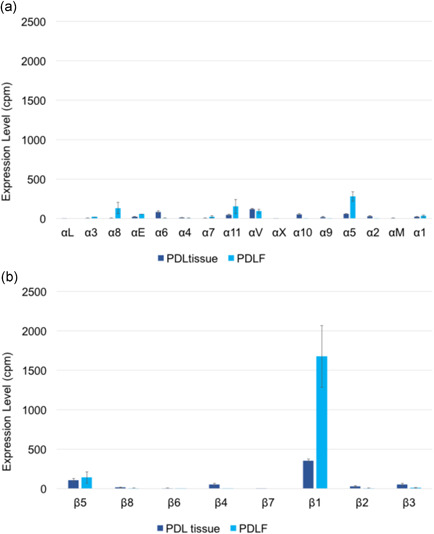
The expression level (cpm) of integrin α (a) and β (b) in PDL tissue and PDLFs showed that integrin β1 was highly expressed in PDL tissue and PDLFs. cpm, counts per million; PDL, periodontal ligament; PDLF, PDL fibroblast

Both PDL tissue and PDLFs contained integrin subunits alpha (α) and beta (β) with different ratios. As shown in Figure 5, integrin α and β were equally expressed in PDL tissue while integrin β was relatively highly expressed in PDLFs. For integrin α subunits, as shown in Figure 6a and Table 8a, αV (24.32%), α6 (16.26%), α5 (12.13%), and α10 (10.72%) were highly expressed in PDL tissue while α5 (34.65%), α11 (18.99%), α8 (16.48%), and αV (11.42%) were highly expressed in PDLFs. For integrin β subunits, as shown in Figure 6b and Table 8b, β1 (PDL tissue: 57.52% and PDLFs: 91.29%) and β5 (PDL tissue: 17.86% and PDLFs: 7.64%) were highly expressed in both PDL tissue and PDLFs.

**Table 8a:** Ratio of integrin β subunit in PDL tissue and PDLFs

Integrin β subunits	PDL tissue (%)	Integrin β subunits	PDLFs (%)
Integrin β1	57.52	Integrin β1	91.29
Integrin β5	17.86	Integrin β5	7.64
Integrin β3	8.68	Integrin β3	0.57
Integrin β4	8.22	Integrin β8	0.34
Integrin β2	4.66	Integrin β2	0.15
Integrin β8	2.48	Integrin β4	0.01
Integrin β6	0.44	Integrin β6	0.001
Integrin β7	0.14	Integrin β7	0.00

Abbreviations: PDL, periodontal ligament; PDLF, PDL fibroblast.

**Table 8b:** Ratio of integrin α subunit in PDL tissue and PDLFs

Integrin α subunits	PDL tissue (%)	Integrin α subunits	PDLFs (%)
Integrin αV	24.32	Integrin α5	34.65
Integrin α6	16.26	Integrin α11	18.99
Integrin α5	12.13	Integrin α8	16.48
Integrin α10	10.72	Integrin αV	11.42
Integrin α11	9.55	Integrin αE	7.02
Integrin α2	5.55	Integrin α1	3.98
Integrin α1	5.02	Integrin α7	2.95
Integrin αE	4.67	Integrin α3	2.63
Integrin α9	3.94	Integrin α6	0.83
Integrin α4	2.08	Integrin α4	0.67
Integrin α8	1.35	Integrin α2	0.27
Integrin α3	1.33	Integrin α10	0.11
Integrin α7	0.93	Integrin α9	0.01
Integrin αM	0.91	Integrin αL	0.00
Integrin αX	0.65	Integrin αX	0.00
Integrin αL	0.59	Integrin αM	0.00

Abbreviations: PDL, periodontal ligament; PDLF, PDL fibroblast.

### 3.4 Western blot analysis

**Figure 7:**
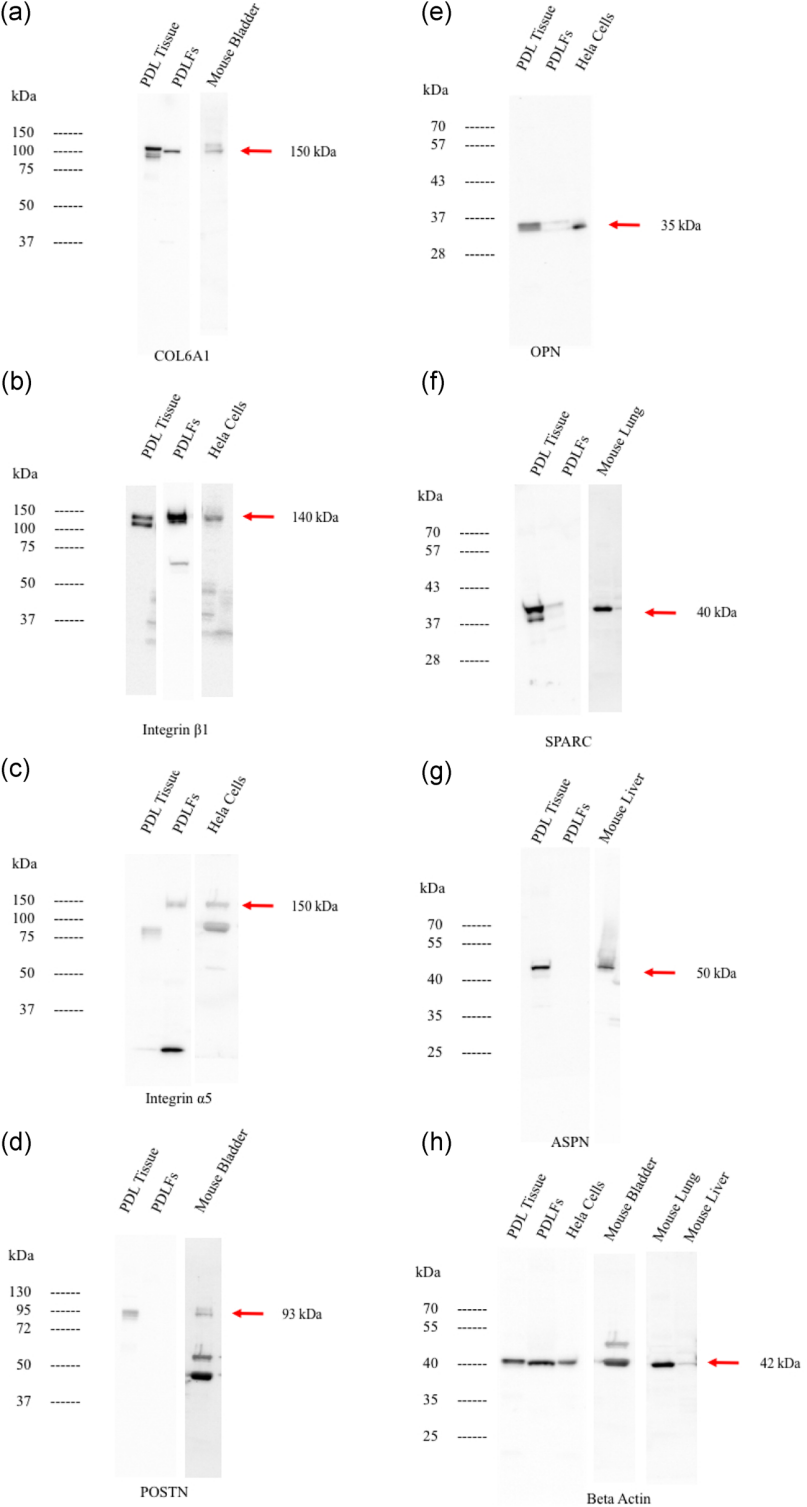
The western blot analysis for collagen type6A1 (a), integrin β1 (b), integrin α5 (c), POSTN (d), OPN (e), SPARC (f), ASPN (g), and beta‐actin (h). Protein expression showed that collagen type6A1 and integrin β1 was highly expressed in both PDL tissue and PDLFs. OPN, POTN, ASPN, and SPARC were highly expressed in PDL tissue while integrin α5 was highly expressed in PDLFs. As positive controls, the extracts from mouse lung, mouse bladder, mouse liver, and Hela cells were utilized in each blotting separately. PDL, periodontal ligament; PDLF, PDL fibroblast

Western blot analysis confirmed protein expression of the genes that were detected by CAGE analysis as mentioned above. In particular, as shown in Figure 7, collagen type VI (COL6A1) was highly expressed in PDL tissue. Integrin β1 was detected in both PDL tissue and PDLFs while integrin α5 was only detected in PDLFs. OPN, ASPN, POSTN, and SPARC were highly detected in PDL tissue but PDLFs.

## 4 DISCUSSION

Our study demonstrated the profiling of the genes to play an important role for structure and function in PDL tissue. These genes can be categorized by their characteristics. We found over 17,000 expressed genes; of these, ECMs, in particular, collagen and noncollagenous ECMs, were the most highly expressed. ECMs play an essential role as biochemical and biomechanical initiators that are required for tissue morphogenesis, differentiation, and homeostasis. ECM also support cell binding and regulate their function such as adhesion and migration. In PDL tissue, ECMs and other related genes also function to maintain the attachment between cementum and alveolar bone, and support daily occlusal loading.

### 4.1 Type VI collagen was highly expressed, next to types I and III collagen, and may support the tight structure of PDL tissue

**Figure 8:**
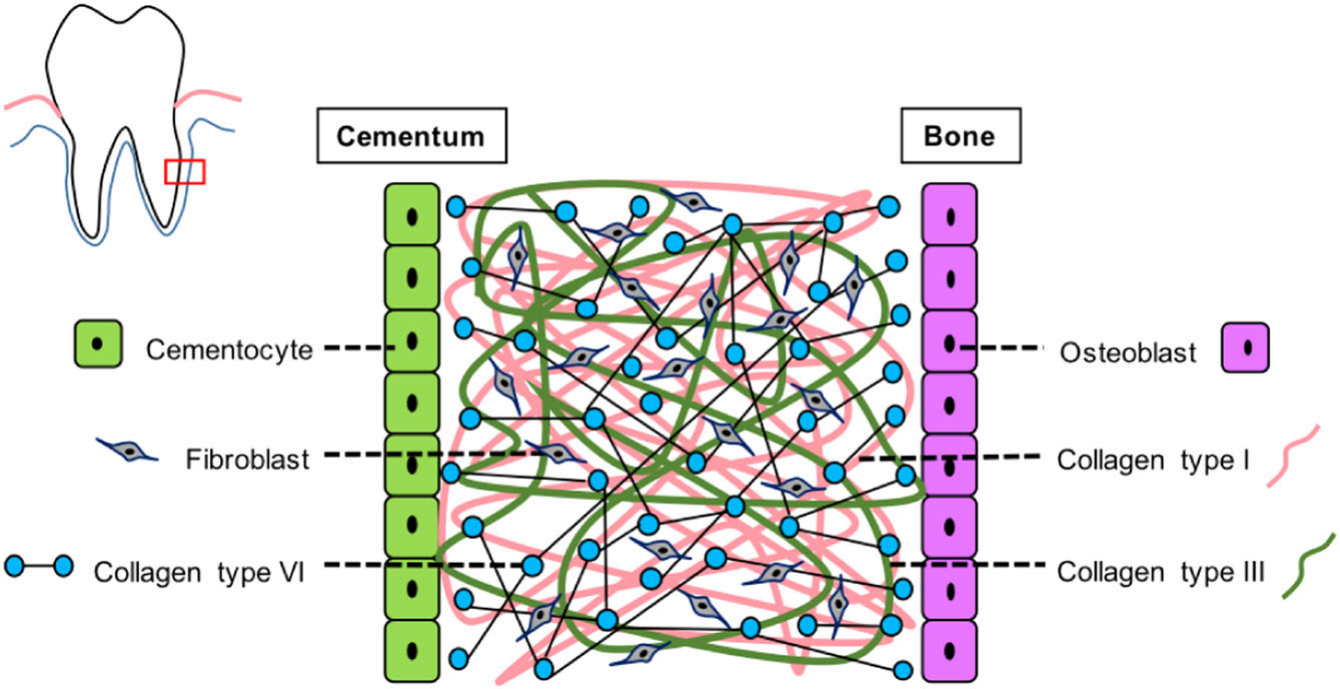
Collagen type VI dispersed in ECMs and bound to other ECMs to make a connection to surrounding connective tissue and arranged their structure including interaction with other collagens. ECM, extracellular matrix

In PDL tissue, collagen types I and III work as the main fibril‐forming collagens to form and stabilize their structure. Unlike other tissue, PDL highly expressed microfibril collagen which was collagen type VI, as shown in Figure 3a. Collagen type VI was distributed in ECMs (Cescon et al., 2015), as shown in Figure 8, and stabilized their structure during absorption of the occlusal force by functioning as an anchor of collagen type I. Previous studies have shown that the absence of collagen type VI may result in dysfunctional regulation of tendon fibrillogenesis (Izu et al., 2011) and affect fibrinogen deposition (Hansen et al., 2012). Apart from being dispersed around ECMs, collagen type VI also binds with other ECMs such as BGN and WARP (Hansen et al., 2012) to make a connection to surrounding connective tissues and arrange their structure. Moreover, other studies have found an interaction between collagen type VI and other collagens such as collagen type II, IV (in basal laminar), and XIV (Bidanset et al., 1992; Bonaldo et al., 1990; Brown et al., 1994; Kuo et al., 1997). Taken together, these results suggest that PDL tissue may require collagen type VI to provide a tight structure and stabilize the PDL from mechanical stress, to prevent tissue destruction. Similar observations have been made in the temporomandibular Joint (data not shown).

### 4.2 POSTN and SPARC were highly expressed in PDL tissue and might support the fibril forming collagen

In Figure 3b, POSTN and SPARC were the major noncollagenous proteins in PDL tissue. POSTN is an ECM that is able to bind to multitargets such as collagen type I and V and integrin αVβ3 and αVβ5 (Zeltz et al., 2014). This type of binding may result in POSTN being able to establish a cross‐linkage and distribution of ECM proteins (Du & Li, 2017). Likewise, SPARC is considered as a bifunctional protein and able to bind to both calcium and collagen type I. These effects may result in SPARC being able to play an essential role in PDL homeostasis and regulate the turnover rate of collagen in PDL tissue (Ribeiro et al., 2014; Trombetta & Bradshaw, 2010). Therefore, these two genes may work together to make a strong structure and support PDL tissue function.

### 4.3 Asporin was the most highly expressed proteoglycan in PDL tissue and may play a major role as a negative regulator for mineralization

Compared to proteoglycans, ASPN, LUM, DCN, and OMD were highly expressed in PDL tissue, as shown in Figure 3c. ASPN suppressed BMP‐2 activity and acted as a negative regulator for PDL mineralization to prevent nonphysiological mineralization such as an ankyloses (Yamada et al., 2007). This character resulted in PDL being an unmineralized tissue despite being surrounded by mineralized structures, and worked as the cushion between cementum and alveolar bone to absorb the mechanical force.

### 4.4 LUM and DCN might be involved in the ECM arrangement while OMD might regulate collagen fibril growth of PDL tissue

DCN and OMD were involved with the structural organizing of ECMs in PDL tissue while LUM bound to collagen type I via a leucine‐rich repeat to organize the collagen fibril (Kalamajski & Oldberg, 2009; Svensson et al., 2000). Similar to LUM, DCN is bound mainly to collagen type I through the leucine‐rich repeat and to collagen type VI at the N terminal region to regulate the normal growth of PDLFs. They organized the collagen fibrils in PDL tissue using these connections (Alimohamad et al., 2005; Hakkinen et al., 2000; Kalamajski et al., 2007). OMD can also bind to collagen type I via a leucine‐rich repeat to control the optimal collagen fibril growth in PDL tissue (Tashima et al., 2018). Taken together, LUM, DCN, and OMD are mainly bound to collagen to regulate and arrange the structure of ECMs.

### 4.5 Integrin β1 was the most highly expressed integrin subunit and might be a major contributor for integrin subunit combination in both PDL tissue and PDLFs

In vertebrates, the integrin family has two different subunits, α and β, and they make a heterodimer to bind to ECMs through an RGD motif (Barczyk et al., 2013, 2010). We identified α and β subunits in both PDL tissue (16 α subunits and 8 β subunits) and PDLFs (13 α subunits and 7 β subunits). As shown in Figure 6a,b, integrin β1 was the most expressed subunit in both PDL tissue and PDLFs and may be considered to be the major component of the heterodimer. Barczyk et al. (2010) showed that integrin β1 was the main β subunit for the possible integrin combination. In our data for the β subunit, β1 was 57.52% and β5 was 17.86% in PDL tissue. Similarly, β1 was 91.29% and β5 was 7.64% in PDLFs. This result implies that the β1 subunit is the dominant subunit in the formation of the heterodimers in PDL tissue and PDLFs. Thus, the most likely combination might be formed by the highest expressed subunits which were αVβ1 in PDL tissue and α5β1 in PDLFs, binding to RGD receptors. Moreover, integrin β5 might possibly be involved in the construction of the integrin αVβ5 combination which also binds to RGD receptors in both PDL tissue and PDLFs. Therefore, RGD receptors might be the main motif to control cell binding affinity in PDL tissue. Other possible combinations such as integrin α6β1 and α10β1 are able to bind to laminin in the basement membrane and collagen fibers. The basement membrane only exists in epithelial cell rests of Malazze and blood vessels in PDL tissue. Thus, the α6 subunit was only slightly expressed in PDLFs, compared to PDL tissue, because of the absence of a basement membrane in cultured PDLFs.

### 4.6 Genes for gene regulation were highly expressed and may be important to promote a high turnover rate in PDL tissue

Besides ECMs, genes that regulate transcription and translation such as MALAT1 and SCARNA2 were highly expressed in PDL tissue as well. MALAT1 is a *trans*‐*acting* factor which regulates alternative splicing of pre‐mRNA by interacting with serine/arginine proteins and influencing their distribution (Gutschner et al., 2013; Tripathi et al., 2010). A previous study found that MALAT1 upregulated fibroblast growth factor 2 which promoted human PDL stem cells proliferation (Chen et al., 2019). SCARNA2, a product of independent transcription, might alter small nucleolar ribonucleoprotein activity and affect the level of modifications within ribosomal RNA (Gerard et al., 2010). Taken together, these data suggest that these genes might promote a high turnover rate in PDL tissue by regulating transcription and translation, including cell proliferation. The turnover rate of collagen in PDL tissue was rapid with a turnover time of 13.5 days, (Orlowski, 1978) comparing to hemin degradation of red blood cells (4 months; Shemin & Rittenberg 1946). Thus, PDL tissue might have a high turnover rate by these factors.

### 4.7 The different gene expression patterns between PDL tissue and PDLFs might provide important information about PDL tissue regeneration

From CAGE data, the highly expressed genes were different between PDL tissue and PDLFs because of the environmental changes (Table 2). However, this data was analyzed from three patients which might cause the heterogenic effect. Therefore, we compared ECM gene expression between PDL tissue and PDLFs that were derived from the same donor (two patients) to avoid genetic heterogeneity. As shown in Tables 6 and 7, TNN, OPN, OCN, OMD, ASPN, and POSTN were remarkably decreased in PDLFs of both patients. Although many genes had decreased in PDLFs, the pattern of decreased genes was similar in both patients. TNN was the most decreased ECM gene in PDLFs, compared to tissue. TNN, also known as tenascin‐W (Barczyk et al., 2013), are glycoproteins that exist in chordates. TNN not only supported cell adhesion and migration but also promoted bone development and angiogenesis, as an adhesion modulatory protein. At the end stage of PDL differentiation, TNN was highly expressed (Nishida et al., 2007). However, TNN disappeared in most adult tissues. On the other hand, a recent study found that TNN was highly expressed in tumor cells and TNN may possibly function as a tumor marker for anticancer therapies (Tucker & Degen, 2019). Thus, TNN may be one of the more important ECMs that promotes cellular attachment in PDL tissue. As mentioned above, we also found other decreased noncollagenous ECMs in PDLFs (ASPN, POSTN, and OMD) although these genes were also highly expressed in PDL tissue. ASPN functioned as a negative regulator for mineralization (Yamada et al., 2007), POSTN supported a strong structure in PDL tissue (Du & Li, 2017), and OMD played an important role for the ECM organization and controlled the optimal growth rate of collagen fibrils (Tashima et al., 2018). These differences may be essential for PDL regeneration.

### 4.8 Two possibilities in the difference of gene profiling between PDL tissue and PDLFs

PDL tissue is considered to be a complex structure that contains not only PDLFs but also other components such as epithelial cell rest of Malassez, blood, and lymph vessels, including those surrounding the hard tissues, cementum, and bone. Each cell expresses different genes which result in different functions in PDL tissue. The combination of gene expression from these cells helps to keep PDL function. When we compared the gene expression patterns between PDL tissue and PDLFs, we noticed that most genes maintained the same expression level but some genes such as TNN, OPN, and OCN were decreased in PDLFs. This result implied two possibilities. One possibility is that the decreased genes may be expressed by the other cells instead of PDLFs. In fact, OPN was found in porcine epithelial cell rests of Malassez and blood vessels. TNN was found in the primary culture of osteoblasts which promotes cell migration and mineralization (Tucker & Degen, 2019). OCN was mainly expressed in osteoblasts (Patti et al., 2013).

The lymphatic vessel was also found in PDL tissue and may be necessary to maintain PDL tissue function (Berggreen & Wiig, 2013; Levy & Bernick 1968). In prior study, TNN expression indicated angiogenesis function by commonly found adjacent to the blood vessel in tumor samples. TNN is also used as a specific marker of glioma‐associated blood vessels and stimulates angiogenesis (Martina et al., 2010) However, we did not find highly expressed lymphatic‐related genes in our study.

On the other hand, TNN, OPN, and OCN were also functionally expressed in PDLFs (Alves et al., 2015; Barczyk et al., 2013) and might be essential genes for PDL function. Another possibility is that the change of environment under culture conditions may affect gene expression levels of ECMs. PDL tissue required a strong structure to support mechanical stress from occlusal loading, promoting bone remodeling in the periodontium, including self‐establishment to maintain their structure while PDLFs do not need to maintain PDL tissue function, only outgrowing themselves on a culture dish.

However, the combination of gene expression from various cells in PDL results the limitation to identify gene expression from each cell type. Further study may perform in situ hybridization, using the specific probe, to detect nucleotide sequences in PDL tissue.

### 4.9 The replenishment of the lost ECM genes may be necessary to recover the essential characteristics of PDL tissue using PDLFs for PDL regeneration

PDLFs were the most abundant cells in PDL tissue and the most compatible cells to provide PDL function. Moreover, it was possible to isolate them in dental procedures such as dental extraction. Therefore, PDLFs were considered to be a priority cell for PDL regeneration. However, cultured PDLFs lost some important genes for PDL tissue function. Thus, the replenishment of lost ECM genes in PDLFs is necessary to recover essential characteristics of PDL tissue. In particular, some highly decreased ECMs such as OPN, ASPN, and POSTN may be critical and may be considered as the candidate genes to restore PDL tissue function. Previous studies have found that OPN expression was increased after mechanical stress stimulation through extracellular signal‐regulated kinase, rho‐kinase pathway, and ATP/P2Y1 in PDLFs (Ito et al., 2014; Wongkhantee et al., 2007, 2008). These results indicated that OPN might respond to mechanical stress and promote remodeling in PDL. According to the molecular structure, OCN contained Gla motif which was able to bind to hydroxyapatite (Razny et al., 2017). This stimulation might partially contribute bone remodeling that surrounds PDL tissue. The replenishment of these genes may recover PDL function during regeneration. Thus, knowledge of the profiling of gene expression in PDL tissue may be critical for the regeneration of PDL tissue in future studies.

## 5 CONCLUSION

Our study is the first study to profile and analyze gene expression of PDL tissue, using second generation sequencing, CAGE. We also compared gene expression between PDL tissue and PDLFs from the same patient for the first time, which is more critical to know the gene profiling between two different environmental conditions. These findings may provide more critical and significant information for PDL tissue reconstruction.

## CONFLICT OF INTERESTS

The authors declare that there are no conflict of interests.

## AUTHOR CONTRIBUTIONS

All authors have made substantial contributions to the conception and design of the study. Nattakarn Hosiriluck, Ayuko Takada, and Haruna Kashiohave been involved in data collection and data analysis. Nattakarn Hosiriluck, Ayuko Takada, Itaru Mizuguchi, and Toshiya Arakawa have been involved in data interpretation, drafting the manuscript, and revising it critically. Nattakarn Hosiriluck and Toshiya Arakawa have given final approval of the version to be published. Toshiya Arakawa supervized the project.

## Supporting information

Supporting information.

## Data Availability

The data that support the finding of this study are available from the first author (Nattakarn Hosiriluck) or the corresponding author (Toshiya Arakawa), upon your request.
