# Supplementary material for: The profiling and analysis of gene expression in human periodontal ligament tissue and fibroblasts
Source: Clin Exp Dent Res. 2022 Feb 1;8(3):658–72. doi: 10.1002/cre2.533 (PMC9209801; doi:10.1002/cre2.533)
Supplement: Supplementary file 1 — Supporting information. [file CRE2-8-658-s001.pdf]

HOSIRILUCK et al.

Gene profiling of human periodontal ligament

The profiling and analysis of gene expression in human periodontal ligament tissue and fibroblasts

Nattakarn Hosiriluck[^1^](#MEP_L_aff1)^,^[^4^](#MEP_L_curr1), Haruna Kashio[^2^](#MEP_L_aff2), Ayuko Takada[^1^](#MEP_L_aff1), Itaru Mizuguchi[^3^](#MEP_L_aff3), Toshiya Arakawa[^1^](#MEP_L_aff1)^,^[^*^](#MEP_L_cor1)0000-0001-7504-353X

^1^Division of Biochemistry, Department of Oral Biology, School of Dentistry, Health Sciences University of Hokkaido, Tobetsu-cho Hokkaido, Japan

^2^Division of Orthodontics and Dentofacial Orthopedics Department of Oral Growth and Development, School of Dentistry, Health Sciences University of Hokkaido, Tobetsu-cho, Hokkaido, Japan

^3^Department of Orthodontics and Dentofacial Orthopedics, Graduate School of Dentistry, Tohoku University, Sendai, Japan

^4^Department of Masticatory Sciences (15^th^ floor), Faculty of Dentistry, Mahidol University, No. 6, Yothi road, Ratchathewi District, Bangkok Thailand 10400

^*^**Correspondence**: Toshiya Arakawa, Division of Biochemistry, Department of Oral Biology, School of Dentistry, Health Sciences University of Hokkaido, Tobetsu-cho, Hokkaido, Japan, Email: arakawa@hoku-iryo-u.ac.jp

01/29/2021; 09/24/2021; 12/28/2021

Abstract

Objectives

The periodontal ligament (PDL) is an important component of periodontium to support dental structure in the alveolar socket. Regeneration of PDL tissue is an effective treatment option for periodontal disease and the profiling of genes involved in this process will be informative. Therefore, our study aims to accurately delineate the profiling of gene expression for PDL tissue regeneration.

Material and methods

We isolated PDL tissues and PDL fibroblasts (PDLFs) from premolar teeth, which were extracted from healthy periodontal status patients undergoing orthodontic treatment. mRNA expression in PDL tissue and PDLFs were analyzed using Cap Analysis Gene Expression (CAGE), which is a second-generation sequencing technique to create profiling. We also determined the protein expression using Western blot.

Results

Collagens (type I, III, and VI), non-collagenous proteins (periostin and osteonectin) and proteoglycans (asporin, lumican, decorin, and osteomodulin) were highly expressed in PDL tissue. Integrin, β1 was also expressed in PDL tissue. On comparison of gene expression between PDL tissue and PDL fibroblasts (PDLFs), 4 PDL marker genes, osteopontin, asporin, periostin and osteonectin, were decreased in PDLFs. The genes for gene regulation were also highly expressed.

Conclusions

Our study demonstrated the overall profiling of mRNA expression in PDL tissue and analyzed the important genes which may be useful for providing specific information for the reconstruction of PDL. We also identified the difference in gene expression between PDL tissue and PDLFs which might provide insights towards PDL regeneration.

Keywords

extracellular matrixes, fibroblasts, periodontal ligament, profiling of gene expression

# supplement

Supporting information.SupMat.pdf

# INTRODUCTION

Periodontium is a critical complex tissue that consists of gingiva, cementum, alveolar bone, and periodontal ligament (PDL). PDL connects the tooth into the alveolar socket by maintaining the attachment between the dental root and bone surface, and provides a cushion for occlusal loading in the masticatory system. Moreover, PDL is able to remodel itself, responding to mechanical stress and maintaining its structure and function under physiologic conditions. (Smith et al. [2019](#MEP_L_bib1); Chukkapalli & Lele, [2018](#MEP_L_bib2)) From a pathological perspective, mechanical overloading may result in the destruction of PDL structure. In current clinical practice, scaling and root planing are commonly performed. However, these treatments are not applicable in severed periodontitis status patients. Thus, PDL regeneration is required as a new treatment option.

Extracellular matrixes (ECMs) are considered as key factors for the tissue repair. In previous studies, collagens, proteoglycans, and non-collagenous proteins were identified as the key ECMs of PDL, (Berkovitz, [1990](#MEP_L_bib5); Butler et al., [1975](#MEP_L_bib3); Dublet et al., [1988](#MEP_L_bib4); Embery, [1990](#MEP_L_bib6)) and most studies had focused on identifying genes from periodontal ligament fibroblasts (PDLFs) instead of tissue because PDLFs are the most abundant cells in PDL tissue. Collagen type I is the major collagen to form the main structure of PDL fiber while other types such as type III are co-distributed with collagen type I. Proteoglycans (e.g. asporin, decorin, and versican(Larjava et al., [1992](#MEP_L_bib7); Yamada et al., [2006](#MEP_L_bib8))) and non-collagenous proteins (e.g. periostin(Xu et al., [2017](#MEP_L_bib9))) have also been reported in PDLFs. However, little is known about overall profiling of gene expression in PDL tissue. According to a comparison of gene expression between PDLFs and PDL tissue, osteopontin was significantly high in PDL tissue. Lallier and Spencer ([2007](#MEP_L_bib10)) Other genes such as pleiotrophin, osteomodulin, alkaline phosphatase, bone sialoprotein 2, periostin, and fibromodulin were slightly high in PDL tissue as well. Based on these results, there has been speculation that the cultured PDLFs may represent an immature cellular form of PDL fibroblasts. (Lallier & Spencer [2007](#MEP_L_bib10); Marchesan et al., [2011](#MEP_L_bib11)) Based on these results, there has been speculation that the cultured PDLFs *in vitro* may present a different nature compared from the PDLFs in PDL tissue under *in vivo*. These different cell condition results the different gene expression. Thus, culturing PDLFs from PDL tissue may alter the ECMs properties due to the environmental changes from *in vivo* to *in vitro*. To regenerate PDL tissue using PDLFs, recovering the originated ECMs *in vivo* must also be considered. Furthermore, epithelial cell rest of Malassez, blood vessel and lymphatic vessel are also present in the PDL tissue area. Including, osteoclasts, osteoblasts and cementoblasts are located in the hard tissue surface. These components may provide molecules such as ECMs in PDL tissue to create a structure and support function. However, the gene profiling only from PDLFs in culture may be altered from environment change and cause insufficient data for the regeneration of PDL tissues. Thus, gene expression in PDL tissue may include genes that are derived from these cells and it may be critical to identify the overall gene expression pattern in PDL tissue. The information of overall gene expression in PDL tissue may be necessary for PDL tissue regeneration.

Our study is the first study to profile and analyze gene expression of PDL tissue, using CAGE method. We analyzed 1) the profiling of whole mRNA expression of PDL tissue by 2^nd^ generation sequencing, 2) the comparison of mRNA expression between PDL tissue and PDLFs from same patient samples.

# MATERIALS AND METHODS

## Sample information

PDL tissue was collected from 8 patients who were required for premolars extraction for orthodontic treatment, purposes under permission by the Ethics Committee, Health Sciences University of Hokkaido (Permission No.135). Our study included only sound teeth with heathy periodontal status. Other condition such as caries, periodontitis, endodontic treated as well as unerupted condition must be excluded. The female patients, age 12-26 years old, were included for this study and all of them are non-smokers. Their identification information has been acquired for this study including, but not limited to, initials, hospital numbers, medical history, surgical history, and dental history. All the information was kept strictly confidential. The patients were de-identified into the code numbers specifically for this study. The patients’ individual information was neither utilized for any purpose other than the purpose of the study nor published in this manuscript. The collected sample from the patients were applied to different methods (S.Table [1](#MEP_L_dmmc1)).

## Materials

Minimum Essential Medium Eagle alpha modification (α-MEM) and Fetal Bovine serum (FBS) were provided from Sigma-Aldrich Co., BambankerTM from Nippon Genetics, Trypsin from Gibco, RNeasy Mini Kit from Qiagen, TRIzolTM Reagent and Reverse Transcription system, from InvirtogenTM, Ex TaqTM from TaKaRa Co. Primers were provided from Hokkaido system science Co., Ltd. and antibodies were obtained from abcam (integrin β1: ab52971, integrin α5: ab150361, osteopontin: ab91655), GeneTex (asporin: GTX104790, periostin: GTX100602, osteonectin: GTX133747, collagen type6A1: GTX109963) and Sigma-Aldrich Co. (beta actin: AC-15), using 1:1000 dilution*.*

## PDLFs isolation from PDL tissue

PDL tissue was collected from teeth that were extracted for orthodontic treatment purposes. The extracted teeth were rinsed with 10% penicillin-streptomycin-added phosphate-buffered saline (PBS). After rinsing, PDL tissue was removed from a tooth at the middle part of the dental root using surgical scalpel blade (No.11). For PDLFs, the PDL tissue was applied directly to a collagen coated culture flask that contained 1▒ml culture medium then the flask was flipped upside-down for 1-2▒hours which caused PDL tissue to attach on the cultured surface of the flask. After the attachment of PDL tissue, the culture flask was flipped back and 3▒ml culture medium was added.

## PDLFs culture

The isolated PDL tissue was cultured in α-MEM with 10% FBS, penicillin/streptomycin (1▒mg/mL), 1% glutamine and amphotericin B (2.5μg/mL) at 37°C with 5% CO_2_. When outgrown to 80% confluence, PDLFs were sub-cultured and frozen with BambankerTM as a stock at 1×10^5^ cells/mL. PDLFs at passage 2–4 were used for experiments. PDLFs were cultured in α-MEM with 10% FBS, penicillin/streptomycin (1▒mg/mL), 1% glutamine and amphotericin B (2.5μg/mL) at 37°C with 5% CO_2_.

## RNA isolation

RNA was isolated from PDL tissue using 1▒mL of TRIzol reagent and homogenized with a homogenizer under instruction. For PDLFs, RNA was isolated using Qiagen, RNeasy Mini Kit. Both RNAs were solubilized with RNase-free water and stored at −80°C. The RNA concentration was measured with NanoDropTM 1000 (Thermo Fisher Scientific).

## Cap Analysis Gene Expression(CAGE)

CAGE, a second-generation sequencing technique that produced a short nucleotide sequence (50 nucleotides) from the 5’ end of messenger RNA in order to determine the gene expression from sample, operated by DNAFORM Co. CAGE was applied to PDL tissue and PDLFs from 4 patients. Patient No.1 and No.2 provided both PDL tissue and PDLFs while Patient No.3 provided only PDL tissue and Patient No.4 provided only PDLFs. The average of gene expression was calculated and measured in counts per million (cpm).

## Polymerase chain reaction (PCR)

Primers of 4 PDL markers (osteopontin, asporin, periostin, and osteonectin) and GAPDH were designed using Primer-BLAST and Primer 3 Plus program as shown in [🏳](#MEP_L_tbl1)Table [1](#MEP_L_tbl1). RNA samples (1 μg) were converted to cDNA using SuperScriptTM II Reverse Transcriptase system. For conventional PCR (cPCR), cDNA (1 μl) were utilized with specific primers and TaKaRa Ex TaqTM for 35 cycles. The PCR products were analyzed by agarose gel electrophoresis. DNA bands were captured with Light-Captured II, Cooled CCD Camera System. For quantitative PCR (qPCR), cDNA (1 μl) was utilized with specific primers, using KAPA SYBR® FAST qPCR Master Mix(2X) Kit. The 2^-ΔΔCt^ value was calculated for fold change of gene expression.

## Protein detection by Western blot

Protein concentrations were measured by spectrophotometry, GeneQuant Pro., using XL-Bradford (APRO SCIENCE, Japan). Protein samples (10 μg) were separated using 5-20% SDS-PAGE gel and transferred to polyvinylidene difluoride (PVDF) membranes. Membranes were blocked with 10% blockA-PBST overnight and incubated with primary antibody for 2▒hours. The protein bands were detected using Enhanced Chemiluminescence (ECL), Immobilon Western Chemiluminescent HRP Substrate (Millipore), and captured with Light-Captured II, Cooled CCD Camera System.

# RESULTS

## Profiling of gene expression in PDL tissue

### Top 20 gene expression in PDL tissue and PDLFs

The top 20 most highly expressed genes in PDL tissue by CAGE method are shown in [🏳](#MEP_L_fig1)Figure [1](#MEP_L_fig1)a, and [🏳](#MEP_L_tbl2)Table [2](#MEP_L_tbl2). In PDL tissue, these genes were able to be categorized into 4 groups: collagens (50.82%), genes for gene regulation (23.27%), non-collagenous ECM (13.73%) and others (12.18%), respectively, as shown in [🏳](#MEP_L_fig2)Figure [2](#MEP_L_fig2)a. In collagen, collagen type I α1 (COL1A1:33.77%), type III α1 (COL3A1:10.35%) and type I α2 (COL1A2:6.69%) were highly expressed in PDL tissue. Genes for gene regulation in PDL tissue were metastasis-associated lung adenocarcinoma transcript 1 (MALAT1:11.16%), small cajal body-specific RNA (SCARNA2:4.52%), humanin-like2 (MTRNR2L2:2.92%), translationally controlled tumor protein (TPT1:1.72%), ribosomal protein lateral stalk subunit P1(RPLP1:1.51%) and ribosomal protein S21 (RPS21:1.44%). Non-collagenous ECM were periostin (POSTN:5.67%), osteonectin (SPARC:5.31%), osteocalcin (OCN:1.44%), and asporin (ASPN:1.31%). Other highly expressed genes in PDL tissue were follicular dendritic cell secreted protein (FDC-SP:3.39%), beta-2-microglobulin (B2M:1.83%), vimentin (VIM:1.70%), S100 calcium-binding protein A6 (S100A6:1.55%), S100 calcium-binding protein A8 (S100A8:1.28%), beta-actin (ACTB:1.23%), and Chemokine (C-X-C motif) ligand 14 (CXCL14:1.18%), respectively. In PDLFs (Table [2](#MEP_L_tbl2)), highly expressed genes were different from PDL tissue which were COL1A1 (15.88%), S100A6 (S100A6:9.72%), MALAT1 (8.87%), Actin Beta (ACTB: 8.4%), Ferritin Heavy Chain1 (FTH1:6.7%).

### Top 20 highly expressed ECM genes in PDL tissue

In Figures [1](#MEP_L_fig1)b and [2](#MEP_L_fig2)b, highly expressed ECMs in PDL tissue were able to be categorized in 3 groups: collagens (75.44%: type I, III, VI, V, XII and XVI, respectively), non-collagenous proteins (19.66%: POSTN, SPARC, OCN, tenascin-N (TNN), osteopontin (OPN), fibronectin (FN1)) and proteoglycans (4.90%: ASPN, lumican (LUM), decorin (DCN), osteomodulin (OMD)).

## Profiling of gene expression in each category of ECMs

### Collagens

In PDL tissue, 27 types of collagens were found to exist. Collagen type I (72.40%: COL1A1 (60.42%), COL1A2 (11.97%)) was the major sub-type and type III (18.52%: COL3A1) was the second, followed by collagens type VI (3.94%: COL6A1 (1.28%), COL6A2 (1.43%), COL6A3 (1.23%)), V (1.62%: COL5A1 (0.76%), COL5A2 (0.77%), COL5A3 (0.09%)), and XII (1.27%: COL12A1), respectively, as shown in [🏳](#MEP_L_fig3)Figure [3](#MEP_L_fig3)a and [🏳](#MEP_L_tbl3a)Tables [3a](#MEP_L_tbl3a) and [3b](#MEP_L_tbl3b). These collagens are able to be categorized by the function which are fibril-forming collagen (type I, III and V), microfibrillar collagen (type VI) and Fibril Associated Collagens with Interrupted Triple helices (FACIT collagen: type XII).

### Non-collagenous proteins

As shown in Figure [3](#MEP_L_fig3)b & [🏳](#MEP_L_tbl4)Table [4](#MEP_L_tbl4), POSTN (37.59%) and SPARC (35.16%) were the two most highly expressed non-collagenous proteins in PDL tissue. Others were OCN (9.54%), TNN (5.27%), OPN (3.68%), FN1 (3.56%), matrix gla protein (MGP; 1.78%), tenascin-C (TNC; 1.39%), bone sialoprotein (IBSP; 0.81%), fibrillin-1 (FBN1; 0.66), dentin matrix acidic phosphoprotein1 (DMP1; 0.48%), desmoplakin (DSP; 0.06%), and Matrix extracellular phosphoglycoprotein (MEPE; 0.02%).

### Proteoglycans

ASPN (30.10%), LUM (26.12%), DCN (14.87%) and OMD (10.74%) were four major proteoglycans in PDL tissue, as shown in Figure [3](#MEP_L_fig3)c & [🏳](#MEP_L_tbl5)Table [5](#MEP_L_tbl5). Others were biglycan (BGN; 4.27%), osteoglycin (OGN; 3.85%), fibromodulin (FMOD; 2.96%), chondroadherin (CHAD; 2.14%), versican (VCAN; 1.51%), aggrecan (ACAN; 1.36%), tsukushi (TSKU; 0.97%), podocan-like protein 1 (PODNL1; 0.63%), and podocan (PODN; 0.49%)

## Comparison of gene expression between PDL tissue and PDLFs

### ECMs

Comparison of the profiling patterns of PDL tissue and PDLFs from 2 patients shows that many ECM genes were decreased in PDLFs compared to PDL tissue. The decreased genes included 4 PDL markers, OPN, POSTN, ASPN, and SPARC, as shown in [🏳](#MEP_L_tbl6)Tables [6](#MEP_L_tbl6) and [7](#MEP_L_tbl7). Quantitative determinations of the 4 PDL markers confirmed these results, as shown in [🏳](#MEP_L_fig4)Figure [4](#MEP_L_fig4).

### Integrins

Both PDL tissue and PDLFs contained integrin subunits alpha (α) and beta (β) with different ratios. As shown in [🏳](#MEP_L_fig5)Figure [5](#MEP_L_fig5), integrin α and β were equally expressed in PDL tissue while integrin β was relatively highly expressed in PDLFs. For integrin α subunits, as shown in [🏳](#MEP_L_fig6)Figure [6](#MEP_L_fig6)a and [🏳](#MEP_L_tbl8a)Table [8a](#MEP_L_tbl8a), αV (24.32%), α6 (16.26%), α5 (12.13%), and α10 (10.72%) were highly expressed in PDL tissue while α5 (34.65%), α11 (18.99%), α8 (16.48%) and αV(11.42%) were highly expressed in PDLFs. For integrin β subunits, as shown in Figure [6](#MEP_L_fig6)b and [🏳](#MEP_L_tbl8b)Table [8b](#MEP_L_tbl8b), β1 (PDL tissue:57.52% and PDLFs:91.29%) and β5 (PDL tissue:17.86% and PDLFs:7.64%) were highly expressed in both PDL tissue and PDLFs.

## Western Blotting

Western Blotting confirmed protein expression of the genes that were detected by CAGE analysis as mentioned above. In particular, as shown in [🏳](#MEP_L_fig7)Figure [7](#MEP_L_fig7), collagen type VI (COL6A1) was highly expressed in PDL tissue. Integrin β1 was detected in both PDL tissue and PDLFs while integrin α5 was only detected in PDLFs. OPN, ASPN, POSTN and SPARC were highly detected in PDL tissue but PDLFs.

# DISCUSSION

Our study demonstrated the profiling of the genes to play an important role for structure and function in PDL tissue. These genes can be categorized by their characteristics. We found over 17,000 expressed genes; of these, ECMs, in particular, collagen and non-collagenous ECMs, were the most highly expressed. ECMs play an essential role as biochemical and biomechanical initiators that are required for tissue morphogenesis, differentiation and homeostasis. ECM also support cell binding and regulate their function such as adhesion and migration. In PDL tissue, ECMs and other related genes also function to maintain the attachment between cementum and alveolar bone, and support daily occlusal loading.

## Type VI collagen was highly expressed, next to types I and III collagen, and may support the tight structure of PDL tissue

In PDL tissue, collagen types I and III work as the main fibril-forming collagens in order to form and stabilize their structure. Unlike other tissue, PDL highly expressed microfibril collagen which was collagen type VI, as shown in Figure [3](#MEP_L_fig3)a. Collagen type VI was distributed in ECMs,(Cescon et al., [2015](#MEP_L_bib12)) as shown in [🏳](#MEP_L_fig8)Figure [8](#MEP_L_fig8), and stabilized their structure during absorption of the occlusal force by functioning as an anchor of collagen type I. Previous studies have shown that absence of collagen type VI may results in dysfunctional regulation of tendon fibrillogenesis(Izu et al., [2011](#MEP_L_bib13)) and affect fibrinogen deposition.Hansen et al. ([2012](#MEP_L_bib14)) Apart from being dispersed around ECMs, collagen type VI also binds with other ECMs such as BGN and WARP(Hansen et al., [2012](#MEP_L_bib14)) to make a connection to surrounding connective tissues and arrange their structure. Moreover, other studies have found an interaction between collagen type VI and other collagens such as collagen type II, IV (in basal laminar) and XIV. (Bonaldo et al. [1990](#MEP_L_bib15); Bidanset et al., [1992](#MEP_L_bib16); Kuo et al., [1997](#MEP_L_bib17); Brown et al., [1994](#MEP_L_bib18)) Taken together, these results suggest that PDL tissue may require collagen type VI to provide a tight structure and stabilize the PDL from mechanical stress, in order to prevent tissue destruction. Similar observations have been made in the Temporomandibular Joint (data not shown).

## POSTN and SPARC were highly expressed in PDL tissue and might support the fibril forming collagen

In Figure [3](#MEP_L_fig3)b, POSTN and SPARC were the major non-collagenous proteins in PDL tissue. POSTN is an ECM that is able to bind to multi-targets such as collagen type I & V and integrin αVβ3 & αVβ5. Zeltz et al. ([2014](#MEP_L_bib19)) This type of binding may result in POSTN being able to establish a cross linkage and distribution of ECM proteins.Du and Li ([2017](#MEP_L_bib20)) Likewise, SPARC is considered as a bifunctional protein and able to bind to both calcium and collagen type I. These effects may result in SPARC being able to play an essential role in PDL homeostasis and regulate the turnover rate of collagen in PDL tissue. (Ribeiro et al. [2014](#MEP_L_bib21); Trombetta & Bradshaw, [2010](#MEP_L_bib22)) Therefore, these two genes may work together to make a strong structure and support PDL tissue function.

## Asporin was the most highly expressed proteoglycan in PDL tissue and may play a major role as a negative regulator for mineralization

Compared to proteoglycans, ASPN, LUM, DCN, and OMD were highly expressed in PDL tissue, as shown in Figure [3](#MEP_L_fig3" \o "Figure 3: The summary of gene profiling of collagens, non-collagenous proteins and proteoglycans in PDL tissue. (a) The ratio of collagens in PDL tissue mainly were collagen type I (72%) and III (19%), followed by type VI (4%), V (2%), XII (1%) and others. (b))c. ASPN suppressed BMP-2 activity and acted as a negative regulator for PDL mineralization in order to prevent non-physiological mineralization such as an ankyloses.Yamada et al. ([2007](#MEP_L_bib23)) This character resulted in PDL being an unmineralized tissue despite being surrounded by mineralized structures, and worked as the cushion between cementum and alveolar bone to absorb the mechanical force.

## LUM and DCN might be involved in the ECM arrangement while OMD might regulate collagen fibril growth of PDL tissue

DCN and OMD were involved with structural organizing of ECMs in PDL tissue while LUM bound to collagen type I via a leucine-rich repeat to organize the collagen fibril. (Kalamajski & Oldberg [2009](#MEP_L_bib24); Svensson et al., [2000](#MEP_L_bib25)) Similar to LUM, DCN bound mainly to collagen type I through the leucine-rich repeat and to collagen type VI at the N terminal region to regulate normal growth of PDLFs. They organized the collagen fibrils in PDL tissue using these connections. (Hakkinen et al. [2000](#MEP_L_bib26); Kalamajski et al., [2007](#MEP_L_bib27); Alimohamad et al., [2005](#MEP_L_bib28)) OMD can also bind to collagen type I via a leucine-rich repeat to control the optimal collagen fibril growth in PDL tissue.Tashima et al. ([2018](#MEP_L_bib29)) Taken together, LUM, DCN and OMD mainly bound to collagen to regulate and arranged the structure of ECMs.

## Integrin β1 was the most highly expressed integrin subunit and might be a major contributor for integrin subunit combination in both PDL tissue and PDLFs

In vertebrates, the integrin family has 2 different subunits, α and β, and they make a heterodimer to bind to ECMs through an RGD motif. (Barczyk et al. [2013](#MEP_L_bib30); Barczyk et al., [2010](#MEP_L_bib31)) We identified α and β subunits in both PDL tissue (16 α subunits and 8 β subunits) and PDLFs (13 α subunits and 7 β subunits). As shown in Figure [6](#MEP_L_fig6)a, b, integrin β1 was the most expressed subunit in both PDL tissue and PDLFs and may be considered to be the major component of the heterodimer. Barczyk et al. showed that integrin β1 was the main β subunit for the possible integrin combination.Barczyk et al. ([2010](#MEP_L_bib31)) In our data for the β subunit, β1 was 57.52% and β5 was 17.86% in PDL tissue. Similarly, β1 was 91.29% and β5 was 7.64% in PDLFs. This result implies that the β1 subunit is the dominant subunit in the formation of the heterodimers in PDL tissue and PDLFs. Thus, the most likely combination might be formed by the highest expressed subunits which were αVβ1 in PDL tissue and α5β1 in PDLFs, binding to RGD receptors. Moreover, integrin β5 might possibly be involved in the construction of integrin αVβ5 combination which also bind to RGD receptors in both PDL tissue and PDLFs. Therefore, RGD receptors might be the main motif to control cell binding affinity in PDL tissue. Other possible combinations such as integrin α6β1and α10β1 are able to bind to laminin in basement membrane and collagen fibers. The basement membrane only exists in epithelial cell rests of Malazze and blood vessels in PDL tissue. Thus, the α6 subunit was only slightly expressed in PDLFs, compared to PDL tissue, because of the absence of a basement membrane in cultured PDLFs.

## Genes for gene regulation were highly expressed and may be important to promote a high turnover rate in PDL tissue

Besides ECMs, genes that regulate transcription and translation such as MALAT1 and SCARNA2 were highly expressed in PDL tissue as well. MALAT1 is a trans-*acting* factor which regulate alternative splicing of pre-mRNA by interacting with serine/arginine proteins and influencing their distribution. (Gutschner et al. [2013](#MEP_L_bib32); Tripathi et al., [2010](#MEP_L_bib33)) A previous study found that MALAT1 upregulated fibroblast growth factor 2 which promoted human PDL stem cells proliferation. Chen et al. ([2019](#MEP_L_bib34)) SCARNA2, a product of independent transcription, might alter small nucleolar ribonucleoprotein (snoRNP) activity and affect the level of modifications within rRNA. Gerard et al. ([2010](#MEP_L_bib35)) Taken together, these data suggest that these genes might promote a high turnover rate in PDL tissue by regulating transcription and translation, including cell proliferation. The turnover rate of collagen in PDL tissue was rapid with turnover time of 13.5 days, (Orlowski, [1978](#MEP_L_bib36)) comparing to hemin degradation of red blood cells (4 months). Shemin and Rittenberg ([1946](#MEP_L_bib37)) Thus, PDL tissue might have a high turnover rate by these factors.

## The different gene expression patterns between PDL tissue and PDLFs might provide important information about PDL tissue regeneration

From CAGE data, the highly expressed genes were different between PDL tissue and PDLFs because of the environment changes (Table [2](#MEP_L_tbl2)). However, this data was analyzed from 3 patients which might cause the heterogenic effect. Therefore, we compared ECM gene expression between PDL tissue and PDLFs that derived from the same donor (2 patients) to avoid genetic heterogeneity. As shown in Tables [6](#MEP_L_tbl6) and [7](#MEP_L_tbl7), TNN, OPN, OCN, OMD, ASPN, and POSTN were remarkably decreased in PDLFs of both patients. Although many genes had decreased in PDLFs, the pattern of decreased genes was similar in both patients. TNN was the most decreased ECM gene in PDLFs, compared to tissue. TNN, also known as tenascin-W(TNW),(Barczyk et al., [2013](#MEP_L_bib30)) are glycoproteins that exists in chordates. TNN not only supported cell adhesion and migration but also promoted bone development and angiogenesis, as an adhesion modulatory protein. At the end stage of PDL differentiation, TNN was highly expressed. Nishida et al. ([2007](#MEP_L_bib38)) However, TNN disappeared in most adult tissues. On the other hand, a recent study found that TNN was highly expressed in tumor cells and TNN may possibly function as a tumor marker for anti-cancer therapies.Tucker and Degen ([2019](#MEP_L_bib39)) Thus, TNN may be one of the more important ECMs that promotes cellular attachment in PDL tissue. As mentioned above, we also found other decreased non-collagenous ECMs in PDLFs (ASPN, POSTN, and OMD) although these genes were also highly expressed in PDL tissue. ASPN functioned as a negative regulator for mineralization,(Yamada et al., [2007](#MEP_L_bib23)) POSTN supported a strong structure in PDL tissue (Du & Li, [2017](#MEP_L_bib20)) and OMD played an important role for the ECM organization and controlled the optimal growth rate of collagen fibrils.Tashima et al. ([2018](#MEP_L_bib29)) These differences may be essential for PDL regeneration.

## Two possibilities in the difference of gene profiling between PDL tissue and PDLFs

PDL tissue is considered to be a complex structure that contains not only PDLFs but also other components such as epithelial cell rest of Malassez, blood and lymph vessels, including those surrounding the hard tissues, cementum and bone. Each cell expresses different genes which result in different functions in PDL tissue. The combination of gene expression from these cells help to keep PDL function. When we compared the gene expression patterns between PDL tissue and PDLFs, we noticed that most genes maintained the same expression level but some genes such as TNN, OPN and OCN were decreased in PDLFs. This result implied two possibilities. One possibility is that the decreased genes may be expressed by the other cells instead of PDLFs. In fact, OPN was found in porcine epithelial cell rests of Malassez and blood vessels. TNN was found in primary culture of osteoblasts which promotes cell migration and mineralization.Tucker & Degen ([2019](#MEP_L_bib39)) OCN was mainly expressed in osteoblasts. Patti et al. ([2013](#MEP_L_bib40))

Lymphatic vessel was also found in PDL tissue and may necessary to maintain PDL tissue function. (Levy & Bernick [1968](#MEP_L_bib41); Berggreen & Wiig, [2013](#MEP_L_bib42)) In prior study, TNN expression indicated angiogenesis function by commonly found adjacent to blood vessel in tumor samples. TNN also used as a specific marker of glioma-associated blood vessels and stimulates angiogenesis.Martina et al. ([2010](#MEP_L_bib43)) However, we did not find highly expressed lymphatic related genes in our study.

On the other hand, TNN, OPN and OCN were also functionally expressed in PDLFs(Alves et al., [2015](#MEP_L_bib44); Barczyk et al., [2013](#MEP_L_bib30)) and might be essential genes for PDL function. Another possibility is that the change of environment under culture conditions may affect gene expression levels of ECMs. PDL tissue required a strong structure to support mechanical stress from occlusal loading, promoting bone remodeling in the periodontium, including self-establishment to maintain their structure while PDLFs do not need to maintain PDL tissue function, only outgrowing themselves on culture dish.

However, the combination of gene expression from various cell in PDL result the limitation to identify gene expression from each cell type. Further study may perform in situ hybridization, using the specific probe, to detect nucleotide sequences in PDL tissue.

## The replenishment of the lost ECM genes may be necessary to recover the essential characteristics of PDL tissue using PDLFs for PDL regeneration

PDLFs were the most abundant cells in PDL tissue and the most compatible cells to provide PDL function. Moreover, it was possible to isolate them in dental procedures such as dental extraction. Therefore, PDLFs were considered to be a priority cell for PDL regeneration. However, cultured PDLFs lost some important genes for PDL tissue function. Thus, the replenishment of lost ECM genes in PDLFs is necessary to recover essential characteristics of PDL tissue. In particular, some highly decreased ECMs such as OPN, ASPN, and POSTN may be critical and may be considered as the candidate genes to restore PDL tissue function. Previous studies have found that OPN expression was increased after mechanical stress stimulation through extracellular signal-regulated kinase, rho-kinase pathway and ATP/P2Y1 in PDLFs. (Wongkhantee et al. [2007](#MEP_L_bib45), [2008](#MEP_L_bib46); Ito et al., [2014](#MEP_L_bib47)) These results indicated that OPN might respond to mechanical stress and promote remodeling in PDL. According to the molecular structure, OCN contained Gla motif which was able to bind to hydroxyapatite.Razny et al. ([2017](#MEP_L_bib48)) This stimulation might partially contribute bone remodeling that surround PDL tissue. The replenishment of these genes may recover PDL function during regeneration. Thus, knowledge of the profiling of gene expression in PDL tissue may be critical for regeneration of PDL tissue in future studies.

# CONCLUSION

Our study is the first study to profile and analyze gene expression of PDL tissue, using 2^nd^ generation sequencing, CAGE. We also compared gene expression between PDL tissue and PDLFs from same patient for the first time, which is more critical to know the gene profiling between 2 different environmental conditions. These findings may provide more critical and significant information for PDL tissue reconstruction.

Acknowledgements

This study was supported in part by Grant-in-aid for the 2018-2019 Research Project of the Research Institute of Health Sciences, Health Sciences University of Hokkaido. Our analytic contribution from Dr. Rie Takai.

# CONFLICT OF INTEREST

The authors declare that there are no conflict of interests.

# AUTHOR CONTRIBUTIONS

All authors have made substantial contributions to conception and design of the study. Nattakarn Hosiriluck [NH], Ayuko Takada [AT] and Haruna Kashio [HK] have been involved in data collection and data analysis. NH, AT, Itaru Mizuguchi, and Toshiya Arakawa [TA] have been involved in data interpretation, drafting the manuscript and revising it critically. NH and TA have given final approval of the version to be published. TA supervised the project.

# openResearch

## Data Availability StatementDataAvailability

The data that support the finding of this study are available from the first author [NH] or the corresponding author [TA], upon your request

References

🛇Alimohamad, H., Habijanac, T., Larjava, H., & Hakkinen, L. (2005). Colocalization of the collagen-binding proteoglycans decorin, biglycan, fibromodulin and lumican with different cells in human gingiva. *Journal of Periodontal Research*, *40*(1), 73–86. 10.1111/j.1600-0765.2004.00776.x

Alves, L. B., Mariguela, V. C., Grisi, M. F., Souza, S. L., Novaes Junior, A. B., Taba Junior, M., Oloveira, P. T., & Palioto, D. B. (2015). Expression of osteoblastic phenotype in periodontal ligament fibroblasts cultured in three-dimensional collagen gel. *Journal of Applied Oral Science*, *23*(2), 206–214. 10.1590/1678-775720140462

🛇Barczyk, M., Bolstad, A. I., & Gullberg, D. (2013). Role of integrins in the periodontal ligament: organizers and facilitators. *Periodontology 2000*, *63*(1), 29–47. 10.1111/prd.12027

☑Barczyk, M., Carracedo, S., & Gullberg, D. (2010). Integrins. *Cell and Tissue Research*, *339*(1), 269–280. 10.1007/s00441-009-0834-6

☑Berggreen, E., & Wiig, H. (2013). Lymphangiogenesis and lymphatic function in periodontal disease. *Journal of Dental Research*, *92*(12), 1074–1080. 10.1177/0022034513504589

☑Berkovitz, B. K. B. (1990). The structure of the periodontal ligament: an update. *The European Journal of Orthodontics*, *12*(1), 51–76.

☑Bidanset, D. J., Guidry, C., Rosenberg, L. C., Choi, H. U., Timpl, R., & Hook, M. (1992). Binding of the proteoglycan decorin to collagen type VI. *Journal of Biological Chemistry*, *267*(8), 5250–5256.

🛇Bonaldo, P., Russo, V., Bucciotti, F., Doliana, R., & Colombatti, A. (1990). Structural and functional features of the alpha 3 chain indicate a bridging role for chicken collagen VI in connective tissues. *Biochemistry*, *29*(5), 1245–1254. 10.1021/bi00457a021

☑Brown, J. C., Golbik, R., Mann, K., & Timpl, R. (1994). Structure and stability of the triple-helical domains of human collagen XIV. *Matrix Biology*, *14*(4), 287–295. 10.1016/0945-053x(94)90194-5

☑Butler, W. T., Birkedal-Hansen, H., Beegle, W. F., Taylor, R. E., & Chung, E. (1975). Proteins of the periodontium. Identification of collagens with the [alpha1(I)]2alpha2 and [alpha1(III)]3 structures in bovine periodontal ligament. *Journal of Biological Chemistry*, *250*(23), 8907–8912.

🛇Cescon, M., Gattazzo, F., Chen, P., & Bonaldo, P. (2015). Collagen VI at a glance. *Journal of Cell Science*, *128*(19), 3525–3531. 10.1242/jcs.169748

🛇Chen, P., Huang, Y., Wang, Y., Li, S., Chu, H., & Rong, M. (2019). MALAT1 overexpression promotes the proliferation of human periodontal ligament stem cells by upregulating fibroblast growth factor 2. *Experimental and Therapeutic Medicine*, *18*(3), 1627–1632. 10.3892/etm.2019.7748

☑Chukkapalli, S. S., & Lele, T. P. (2018). Periodontal cell mechanotransduction. *Open Biology*, *8*(9), 10.1098/rsob.180053

☑Du, J., & Li, M. (2017). Functions of Periostin in dental tissues and its role in periodontal tissues' regeneration. *Cellular and Molecular Life Science*, *74*(23), 4279–4286. 10.1007/s00018-017-2645-3

Dublet, B., Dixon, E., de Miguel, E., van der Rest, M. (1988). Bovine type XII collagen: amino acid sequence of a 10 kDa pepsin fragment from periodontal ligament reveals a high degree of homology with the chicken alpha 1(XII) sequence. *FEBS Letters*, *233*(1), 177–180. 10.1016/0014-5793(88)81379-6

☑Embery, G. (1990). An update on the biochemistry of the periodontal ligament. *European Journal of Orthodontics*, *12*(1), 77–80. 10.1093/ejo/12.1.77

☑Gerard, M. A., Myslinski, E., Chylak, N., Baudrey, S., Krol, A., & Carbon, P. (2010). The scaRNA2 is produced by an independent transcription unit and its processing is directed by the encoding region. *Nucleic Acids Research*, *38*(2), 370–381. 10.1093/nar/gkp988

🛇Gutschner, T., Hammerle, M., Eissmann, M., Hsu, J., Kim, Y., Hung, G., Revenko, A., Arun, G., Stentrup, M., Gross, M., Zörnig, M., Macleod, A. R., Spector, D. L., & Diederichs, S. (2013). The noncoding RNA MALAT1 is a critical regulator of the metastasis phenotype of lung cancer cells. *Cancer Research*, *73*(3), 1180–1189. 10.1158/0008-5472.CAN-12-2850

🛇Hakkinen, L., Strassburger, S., Kahari, V. M., Scott, P. G., Eichstetter, I., Lozzo, R. V., & Larjava, H. (2000). A role for decorin in the structural organization of periodontal ligament. *Laboratory Investigation*, *80*(12), 1869–1880. 10.1038/labinvest.3780197

🛇Hansen, U., Allen, J. M., White, R., Moscibrocki, C., Bruckner, P., Bateman, J. F., & Fitzgerald, J. (2012). WARP interacts with collagen VI-containing microfibrils in the pericellular matrix of human chondrocytes. *PLoS One*, *7*(12), e52793. 10.1371/journal.pone.0052793

Ito, M., Arakawa, T., Okayama, M., Shitara, A., Mizoguchi, I., & Takuma, T. (2014). Gravity loading induces adenosine triphosphate release and phosphorylation of extracellular signal-regulated kinases in human periodontal ligament cells. *J Investig Clin Dent*, *5*(4), 266–274. 10.1111/jicd.12049

☑Izu, Y., Ansorge, H. L., Zhang, G., Soslowsky, L. J., Bonaldo, P., Chu, M. L., & Birk, D. E. (2011). Dysfunctional tendon collagen fibrillogenesis in collagen VI null mice. *Matrix Biology*, *30*(1), 53–61. 10.1016/j.matbio.2010.10.001

☑Kalamajski, S., Aspberg, A., & Oldberg, A. (2007). The decorin sequence SYIRIADTNIT binds collagen type I. *Journal of Biological Chemistry*, *282*(22), 16062–16067. 10.1074/jbc.M700073200

☑Kalamajski, S., & Oldberg, A. (2009). Homologous sequence in lumican and fibromodulin leucine-rich repeat 5-7 competes for collagen binding. *Journal of Biological Chemistry*, *284*(1), 534–539. 10.1074/jbc.M805721200

☑Kuo, H. J., Maslen, C. L., Keene, D. R., & Glanville, R. W. (1997). Type VI collagen anchors endothelial basement membranes by interacting with type IV collagen. *Journal of Biological Chemistry*, *272*(42), 26522–26529. 10.1074/jbc.272.42.26522

🛇Lallier, T. E., & Spencer, A. (2007). Use of microarrays to find novel regulators of periodontal ligament fibroblast differentiation. *Cell and Tissue Research*, *327*(1), 93–109. 10.1007/s00441-006-0282-5

☑Larjava, H., Hakkinen, L., & Rahemtulla, F. (1992). A biochemical analysis of human periodontal tissue proteoglycans. *Biochemical Journal*, *284*(*Pt 1*), 267–274. 10.1042/bj2840267

☑Levy, B. M., & Bernick, S. (1968). Studies on the biology of the periodontium of marmosets: V. Lymphatic vessels of the periodontal ligament. *Journal of Dental Research*, *47*(6), 1166–1170. 10.1177/00220345680470062601

☑Marchesan, J. T., Scanlon, C. S., Soehren, S., Matsuo, M., & Kapila, Y. L. (2011). Implications of cultured periodontal ligament cells for the clinical and experimental setting: a review. *Archives of Oral Biology*, *56*(10), 933–943. 10.1016/j.archoralbio.2011.03.003

🛇Martina, E., Degen, M., Ruegg, C., Merlo, A., Lino, M. M., Chiquet‐Ehrismann, R., & Brellier, F. (2010). Tenascin‐W is a specific marker of glioma‐associated blood vessels and stimulates angiogenesis in vitro. *The FASEB Journal*, *24*(3), 778–787. 10.1096/fj.09-140491

☑Nishida, E., Sasaki, T., Ishikawa, S. K., Kosaka, K., Aino, M., Noguchi, T., Teranaka, T., Shimizu, N., & Saito, M. (2007). Transcriptome database KK-Periome for periodontal ligament development: expression profiles of the extracellular matrix genes. *Gene*, *404*(1-2), 70–79. 10.1016/j.gene.2007.09.009

☑Orlowski, W. A. (1978). Biochemical studies of collagen turnover in rat incisor periodontal ligament. *Archives of Oral Biology*, *23*(12), 1163–1165. 10.1016/0003-9969(78)90125-5

🛇Patti, A., Gennari, L., Merlotti, D., Dotta, F., & Nuti, R. (2013). Endocrine actions of osteocalcin. *International Journal of Endocrinology*, *2013*, 846480. 10.1155/2013/846480

🛇Razny, U., Fedak, D., Kiec-Wilk, B., Goralska, J., Gruca, A., Zdzienicka, A., Kiec-Klimczak, M., Solnica, B., Hubalewska-Dydejczyk, A., & Malczewska-Malec, M. (2017). Carboxylated and undercarboxylated osteocalcin in metabolic complications of human obesity and prediabetes. *Diabetes/Metabolism Research and Reviews*, *33*(3), 10.1002/dmrr.2862

🛇Ribeiro, N., Sousa, S. R., Brekken, R. A., & Monteiro, F. J. (2014). Role of SPARC in bone remodeling and cancer-related bone metastasis. *Journal of Cellular Biochemistry*, *115*(1), 17–26. 10.1002/jcb.24649

☑Shemin, D., & Rittenberg, D. (1946). The life span of the human red blood cell. *Journal of Biological Chemistry*, *166*(2), 627–636.

⮽Smith, P. C., Martinez, C., Martinez, J., & McCulloch, C. A. (2019). Role of Fibroblast Populations in Periodontal Wound Healing and Tissue Remodeling. *Frontiers in Physiology*, *10*, 270–280. 10.3389/fphys.2019.00270

☑Svensson, L., Narlid, I., & Oldberg, A. (2000). Fibromodulin and lumican bind to the same region on collagen type I fibrils. *FEBS Letters*, *470*(2), 178–182. 10.1016/s0014-5793(00)01314-4

🛇Tashima, T., Nagatoishi, S., Caaveiro, J. M. M., Nakakido, M., Sagara, H., Kusano-Arai, O., Iwanari, H., Mimuro, H., Hamakubo, T., Ohnuma, S., & Tsumoto, K. (2018). Molecular basis for governing the morphology of type-I collagen fibrils by Osteomodulin. *Commun Biol*, *1*, 33. 10.1038/s42003-018-0038-2

☑Tripathi, V., Ellis, J. D., Shen, Z., Song, D. Y., Pan, Q., Watt, A. T., Freier, S. M., Bennett, C. F., Sharma, A., Bubulya, P. A., Blencowe, B. J., Prasanth, S. G., & Prasanth, K. V. (2010). The nuclear-retained noncoding RNA MALAT1 regulates alternative splicing by modulating SR splicing factor phosphorylation. *Molecular Cell*, *39*(6), 925–938. 10.1016/j.molcel.2010.08.011

🛇Trombetta, J. M., & Bradshaw, A. D. (2010). SPARC/osteonectin functions to maintain homeostasis of the collagenous extracellular matrix in the periodontal ligament. *Journal of Histochemistry and Cytochemistry*, *58*(10), 871–879. 10.1369/jhc.2010.956144

🛇Tucker, R. P., & Degen, M. (2019). The Expression and Possible Functions of Tenascin-W During Development and Disease. *Frontiers in Cell and Developmental Biology*, *7*, 53. 10.3389/fcell.2019.00053

☑Wongkhantee, S., Yongchaitrakul, T., & Pavasant, P. (2007). Mechanical stress induces osteopontin expression in human periodontal ligament cells through rho kinase. *Journal of Periodontology*, *78*(6), 1113–1119. 10.1902/jop.2007.060433

☑Wongkhantee, S., Yongchaitrakul, T., & Pavasant, P. (2008). Mechanical stress induces osteopontin via ATP/P2Y1 in periodontal cells. *Journal of Dental Research*, *87*(6), 564–568. 10.1177/154405910808700601

☑Xu, H. Y., Nie, E. M., Deng, G., Lai, L. Z., Sun, F. Y., Tian, H., Fang, F. C., Zou, Y. G., Wu, B. L., & Ou-Yang, J. (2017). Periostin is essential for periodontal ligament remodeling during orthodontic treatment. *Molecular Medicine Reports*, *15*(4), 1800–1806. 10.3892/mmr.2017.6200

☑Yamada, S., Ozawa, Y., Tomoeda, M., Matoba, R., Matsubara, K., & Murakami, S. (2006). Regulation of PLAP-1 expression in periodontal ligament cells. *Journal of Dental Research*, *85*(5), 447–451. 10.1177/154405910608500510

☑Yamada, S., Tomoeda, M., Ozawa, Y., Yoneda, S., Terashima, Y., Ikezawa, K., Ikegawa, S., Saito, M., Toyosawa, S., & Murakami, S. (2007). PLAP-1/asporin, a novel negative regulator of periodontal ligament mineralization. *Journal of Biological Chemistry*, *282*(32), 23070–23080. 10.1074/jbc.M611181200

🛇Zeltz, C., Orgel, J., & Gullberg, D. (2014). Molecular composition and function of integrin-based collagen glues-introducing COLINBRIs. *Biochimica et Biophysica Acta/General Subjects*, *1840*(8), 2533–2548. 10.1016/j.bbagen.2013.12.022

Figure 1: CAGE data reported (a) the expression level of top 20 most expressed genes in PDL tissue (Y axis shows the expression level as counts per millions (cpm)) and (b) important ECM genes that are highly expressed in PDL tissue (Y axis refers to gene expression level (cpm)).

Figure 2: The summary of gene profiling by categories in PDL tissue. (a) Ratio of 4 categories in all top 20 genes: collagens (51%), genes for gene regulation (23%), non-collagenous ECM (14%) and others (12%). (b) Ratio of 3 categories in top 20 ECM genes: collagens (75%), non-collagenous proteins (20%) and proteoglycans (5%)

Figure 3: The summary of gene profiling of collagens, non-collagenous proteins and proteoglycans in PDL tissue. (a) The ratio of collagens in PDL tissue mainly were collagen type I (72%) and III (19%), followed by type VI (4%), V (2%), XII (1%) and others. (b) Ratio of non-collagenous proteins in PDL tissue mainly were POSTN (38%) and SPARC (35%). (c) Ratio of proteoglycan in PDL tissue mainly were ASPN (30%), LUM (26%), DCN (15%) and OMD (11%).

Figure 4: Comparison of gene expression between PDL tissue and PDLFs by quantitative PCR in 4 PDL marker genes (OPN, ASPN, POSTN and SPARC).

Figure 5: The ratio of integrin α and β in PDL tissue and PDLFs. Integrin α and β were equally expressed in PDL tissue while integrin β was relatively highly expressed in PDLFs.

Figure 6: The expression level (cpm) of integrin α (a) and β (b) in PDL tissue and PDLFs showed that integrin β1 was highly expressed in PDL tissue and PDLFs.

Figure 7: The western blotting for collagen type6A1 (7a), Integrin β1 (7b), Integrin α5 (7c), POSTN (7d), OPN (7e), SPARC (7▒f), ASPN (7▒g) and Beta Actin (7▒h). Protein expression showed that collagen type6A1 and integrin β1 was highly expressed in both PDL tissue and PDLFs. OPN, POTN, ASPN and SPARC were highly expressed in PDL tissue while integrin α5 was highly expressed in PDLFs. As positive controls, the extracts from mouse lung, mouse bladder, mouse liver, and Hela cells were utilized in each blotting separately.

Figure 8: Collagen type VI dispersed in ECMs and bound to other ECMs to make a connection to surrounding connective tissue and arranged their structure including interaction with other collagens.

Table 1 Primer sets of 4 PDL markers and GAPDH

| Gene | Primer Sequence | | Product Size(bp) |
| --- | --- | --- | --- |
| OPN (NM_001040058.2) | Forward | ACCCATCTCAGAAGCAGAATCTCC | 462 |
|  | Reverse | CACCATTCAACTCCTCGCTTTCC |  |
| ASPN (NM_017680.5) | Forward | CTTTGTGCTCTGCCAAACCC | 440 |
|  | Reverse | GGACAGATACAGCCTTCGCA |  |
| POSTN (NM_006475.3) | Forward | GTCTTTGAGACGCTGGAAGG | 201 |
|  | Reverse | CAAGATCCGTGAAGGTGGTT |  |
| SPARC (NM_003118.4) | Forward | GGAAGAAACTGTGGCAGAGGTGA | 469 |
|  | Reverse | TGTTGTCCTCATCCCTCTCATAC |  |
| GAPDH (NM_002046.7) | Forward | GAGAAGGCTGGGGCTCATTT | 231 |
|  | Reverse | AGTGATGGCATGGACTGTGG |  |

Table 2 Percentage of top 20 genes in PDL tissue and PDLFs

| Top 20 genes in PDL tissue | Percentage | Top 20 genes in PDLFs | Percentage |
| --- | --- | --- | --- |
| COL1A1 | 33.77 | COL1A1 | 15.88 |
| MALAT1 | 11.16 | S100A6 | 9.72 |
| COL3A1 | 10.35 | MALAT1 | 8.87 |
| COL1A2 | 6.69 | ACTB | 8.40 |
| POSTN | 5.67 | FTH1 | 6.70 |
| SPARC | 5.31 | TMSB10 | 5.58 |
| SCARNA2 | 4.52 | TMSB4X | 4.67 |
| FDCSP | 3.39 | VIM | 4.25 |
| MTRNR2L2 | 2.92 | ACTG1 | 4.15 |
| B2M | 1.83 | COL1A2 | 3.46 |
| TPT1 | 1.72 | TPT1 | 3.38 |
| VIM | 1.70 | RPLP1 | 3.15 |
| S100A6 | 1.55 | GAPDH | 3.11 |
| RPLP1 | 1.51 | LGALS1 | 2.92 |
| RPS21 | 1.44 | MYL6 | 2.79 |
| OCN | 1.44 | MYL6B | 2.73 |
| ASPN | 1.31 | RP11 | 2.72 |
| S100A8 | 1.28 | MTRNR2L2 | 2.53 |
| ACTB | 1.23 | RPS21 | 2.50 |
| CXCL14 | 1.18 | FTL | 2.47 |

Table 3a Types of collagens in PDL tissue

| No. | Collagen Type | Percentage | No. | Collagen Type | Percentage |
| --- | --- | --- | --- | --- | --- |
| 1 | COL1 | 72.40 | 15 | COL17 | 0.05 |
| 2 | COL3 | 18.52 | 16 | COL8 | 0.05 |
| 3 | COL6 | 3.94 | 17 | COL27 | 0.05 |
| 4 | COL5 | 1.62 | 18 | COL9 | 0.04 |
| 5 | COL12 | 1.27 | 19 | COL24 | 0.03 |
| 6 | COL16 | 0.56 | 20 | COL10 | 0.02 |
| 7 | COL11 | 0.43 | 21 | COL26 | 0.01 |
| 8 | COL4 | 0.39 | 22 | COL25 | 0.01 |
| 9 | COL18 | 0.14 | 23 | COL28 | 0.01 |
| 10 | COL13 | 0.11 | 24 | COL23 | 0.004 |
| 11 | COL21 | 0.11 | 25 | COL22 | 0.004 |
| 12 | COL14 | 0.09 | 26 | COL2 | 0.004 |
| 13 | COL7 | 0.09 | 27 | COL20 | 0.001 |
| 14 | COL15 | 0.06 |  | | |

Table 3b All collagens in PDL tissue

| Collagens | Percentage | Collagens | Percentage |
| --- | --- | --- | --- |
| COL1A1 | 60.42 | COL17A1 | 0.05 |
| COL3A1 | 18.52 | COL27A1 | 0.05 |
| COL1A2 | 11.97 | COL4A3BP | 0.03 |
| COL6A2 | 1.43 | COL24A1 | 0.03 |
| COL6A1 | 1.28 | COL8A2 | 0.03 |
| COL12A1 | 1.27 | COL9A2 | 0.02 |
| COL6A3 | 1.23 | COL10A1 | 0.02 |
| COL5A2 | 0.77 | COL8A1 | 0.02 |
| COL5A1 | 0.76 | COL9A3 | 0.01 |
| COL16A1 | 0.56 | COL26A1 | 0.01 |
| COL11A1 | 0.42 | COL25A1 | 0.01 |
| COL4A1 | 0.18 | COL4A5 | 0.01 |
| COL4A2 | 0.17 | COL28A1 | 0.01 |
| COL18A1 | 0.14 | COL23A1 | 0.004 |
| COL13A1 | 0.11 | COL11A2 | 0.004 |
| COL21A1 | 0.11 | COL2A1 | 0.004 |
| COL14A1 | 0.09 | COL22A1 | 0.004 |
| COL5A3 | 0.09 | COL20A1 | 0.001 |
| COL7A1 | 0.08 | COL4A4 | 0.001 |
| COL15A1 | 0.06 |  | |

Table 4 Non-collagenous proteins in PDL tissue

| Noncollagenous proteins | Percentage |
| --- | --- |
| POSTN | 37.59 |
| SPARC | 35.16 |
| OCN | 9.54 |
| TNN | 5.27 |
| OPN | 3.68 |
| FN1 | 3.56 |
| MGP | 1.78 |
| TNC | 1.39 |
| IBSP | 0.81 |
| FBN1 | 0.66 |
| DMP1 | 0.48 |
| DSP | 0.06 |
| MEPE | 0.02 |

Table 5 Proteoglycans in PDL tissue

| Proteoglycans | Percentage |
| --- | --- |
| ASPN | 30.10 |
| LUM | 26.12 |
| DCN | 14.87 |
| OMD | 10.74 |
| BGN | 4.27 |
| OGN | 3.85 |
| FMOD | 2.96 |
| CHAD | 2.14 |
| VCAN | 1.51 |
| ACAN | 1.36 |
| TSKU | 0.97 |
| PODNL1 | 0.63 |
| PODN | 0.49 |

Table 6 Expression of ECM genes in PDL tissue and PDLFs of patient 1

| Gene | PDL tissue | PDLFs | Fold change |
| --- | --- | --- | --- |
| TNN | 1320.48 | 0.08 | 16506.00 |
| OPN | 855.48 | 0.08 | 10693.50 |
| OCN | 1529.36 | 0.20 | 7646.80 |
| OMD | 663.68 | 0.88 | 754.18 |
| ASPN | 1714.12 | 2.84 | 603.56 |
| POSTN | 6969.29 | 187.12 | 37.25 |
| LUM | 1847.72 | 71.28 | 25.92 |
| COL3A1 | 14993.11 | 1416.92 | 10.58 |
| COL12A1 | 1144.68 | 192.84 | 5.94 |
| COL16A1 | 372.40 | 92.16 | 4.04 |
| COL5A1 | 411.44 | 205.44 | 2.00 |
| SPARC | 5617.65 | 3121.61 | 1.80 |
| COL1A2 | 8218.90 | 5108.33 | 1.61 |
| COL1A1 | 40956.32 | 26045.81 | 1.57 |
| COL6A3 | 1057.44 | 718.52 | 1.47 |
| DCN | 886.48 | 621.44 | 1.43 |
| COL5A2 | 518.40 | 596.60 | 0.87 |
| COL6A2 | 1049.96 | 1864.96 | 0.56 |
| FN1 | 772.84 | 1752.60 | 0.44 |
| COL6A1 | 1155.00 | 2816.69 | 0.41 |

Table 7 Expression of ECM genes in PDL tissue and PDLFs of patient 2

| Gene | PDL tissue | PDLFs | Fold change |
| --- | --- | --- | --- |
| TNN | 1058.64 | 0.12 | 8822.00 |
| OPN | 1425.16 | 0.17 | 8383.29 |
| OCN | 3122.53 | 0.75 | 4163.37 |
| OMD | 871.40 | 33.94 | 25.67 |
| POSTN | 10789.34 | 617.24 | 17.48 |
| ASPN | 2533.45 | 174.80 | 14.49 |
| COL3A1 | 9815.38 | 1043.93 | 9.40 |
| LUM | 1670.60 | 239.27 | 6.98 |
| COL16A1 | 353.08 | 64.76 | 5.45 |
| COL5A1 | 856.88 | 316.78 | 2.70 |
| SPARC | 6827.89 | 4209.33 | 1.62 |
| COL6A3 | 734.36 | 478.09 | 1.54 |
| COL5A2 | 628.72 | 424.49 | 1.48 |
| COL1A1 | 29131.86 | 22852.53 | 1.27 |
| COL1A2 | 7665.50 | 6315.72 | 1.21 |
| COL12A1 | 726.52 | 681.38 | 1.07 |
| DCN | 1089.04 | 1480.45 | 0.74 |
| COL6A2 | 1144.04 | 2259.99 | 0.51 |
| COL6A1 | 840.80 | 2968.94 | 0.28 |
| FN1 | 1063.04 | 5678.03 | 0.19 |

Table 8a Ratio of integrin β subunit in PDL tissue and PDLFs

| Integrin β subunits | PDL tissue (%) | Integrin β subunits | PDLFs (%) |
| --- | --- | --- | --- |
| Integrin β1 | 57.52 | Integrin β1 | 91.29 |
| Integrin β5 | 17.86 | Integrin β5 | 7.64 |
| Integrin β3 | 8.68 | Integrin β3 | 0.57 |
| Integrin β4 | 8.22 | Integrin β8 | 0.34 |
| Integrin β2 | 4.66 | Integrin β2 | 0.15 |
| Integrin β8 | 2.48 | Integrin β4 | 0.01 |
| Integrin β6 | 0.44 | Integrin β6 | 0.001 |
| Integrin β7 | 0.14 | Integrin β7 | 0.00 |

Table 8b Ratio of integrin α subunit in PDL tissue and PDLFs

| Integrin α subunits | PDL tissue (%) | Integrin α subunits | PDLFs (%) |
| --- | --- | --- | --- |
| Integrin αV | 24.32 | Integrin α5 | 34.65 |
| Integrin α6 | 16.26 | Integrin α11 | 18.99 |
| Integrin α5 | 12.13 | Integrin α8 | 16.48 |
| Integrin α10 | 10.72 | Integrin αV | 11.42 |
| Integrin α11 | 9.55 | Integrin αE | 7.02 |
| Integrin α2 | 5.55 | Integrin α1 | 3.98 |
| Integrin α1 | 5.02 | Integrin α7 | 2.95 |
| Integrin αE | 4.67 | Integrin α3 | 2.63 |
| Integrin α9 | 3.94 | Integrin α6 | 0.83 |
| Integrin α4 | 2.08 | Integrin α4 | 0.67 |
| Integrin α8 | 1.35 | Integrin α2 | 0.27 |
| Integrin α3 | 1.33 | Integrin α10 | 0.11 |
| Integrin α7 | 0.93 | Integrin α9 | 0.01 |
| Integrin αM | 0.91 | Integrin αL | 0.00 |
| Integrin αX | 0.65 | Integrin αX | 0.00 |
| Integrin αL | 0.59 | Integrin αM | 0.00 |
